# Source reservoir controls on the size, frequency, and composition of large-scale volcanic eruptions

**DOI:** 10.1126/sciadv.add1595

**Published:** 2024-05-10

**Authors:** Catherine A. Booth, Matthew D. Jackson, R. Stephen J. Sparks, Alison C. Rust

**Affiliations:** ^1^ Department of Earth Science and Engineering, Imperial College London, London, UK.; ^2^ School of Earth Sciences, University of Bristol, Bristol, UK.

## Abstract

Large-scale, explosive volcanic eruptions are one of the Earth’s most hazardous natural phenomena. We demonstrate that their size, frequency, and composition can be explained by processes in long-lived, high-crystallinity source reservoirs that control the episodic creation of large volumes of eruptible silicic magma and its delivery to the subvolcanic chamber where it is stored before eruption. Melt percolates upward through the reservoir and accumulates a large volume of low-crystallinity silicic magma which remains trapped until buoyancy causes magma-driven fractures to propagate into the overlying crust, allowing rapid magma transfer from the reservoir into the chamber. Ongoing melt percolation in the reservoir accumulates a new magma layer and the process repeats. Our results suggest that buoyancy, rather than crystallinity, is the key control on magma delivery from the source reservoir. They identify an optimum reservoir size for the largest silicic eruptions that is consistent with data from natural systems and explain why larger magnitude eruptions are not observed on Earth.

## Teaser

Magma accumulates in long-lived, high crystallinity source reservoirs to supply the Earth’s most hazardous volcanic eruptions.

## INTRODUCTION

Very large magnitude explosive eruptions (*M* > 7) expel tens to thousands of cubic kilometers of silicic magma (*1*). Their global frequency is inversely proportional to the volume of magma released; the largest eruptions recur over timescales of order 100’s ka (*1*, *2*). These super-eruptions are rare but have a global impact on the environment and human populations (*3*). Fundamental questions remain concerning the underlying processes that control the accumulation and eruption of such large volumes of magma and the maximum eruption size.

**Fig. 1.**
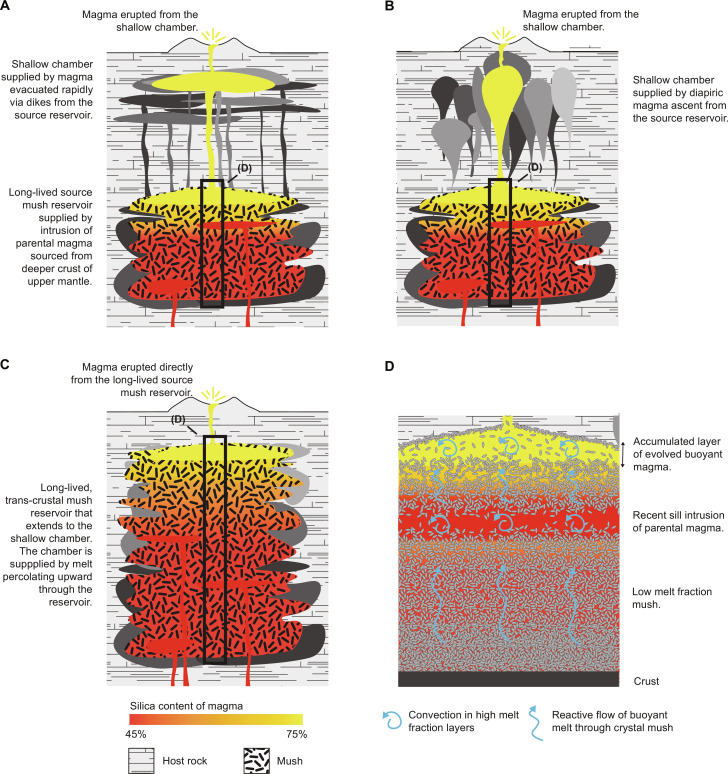
Source reservoir processes that may supply a large volcanic eruption. A long-lived, high crystallinity mush reservoir is created by intrusion of parental magma sourced from the deep crust or upper mantle. Low-crystallinity magma formed in the reservoir can evacuate and supply a shallow chamber via (**A**) dikes or (**B**) diapirs; alternatively, the reservoir can span the crust, and melt can be supplied direct to a shallow chamber (**C**). Reactive, percolative flow of melt through the source reservoir accumulates a layer of evolved magma near the top of the reservoir (**D**).

Crystal-specific geochemical and petrological data reveal contrasting depths and timescales for magma accumulation and storage. Pre-eruption storage is consistently interpreted at ~3 to 8 km depth (Fig. 1) (*4*–*6*). Accumulation timescales in these shallow chambers are interpreted to span 10s of a to 10s of ka (*6*–*9*). The chambers represent only the shallowest portions of vertically extensive magmatic systems that are ultimately created and maintained by the intrusion of mantle-derived basalt (*10*–*12*). The deeper parts of these systems are long-lived, with interpreted timescales of magma accumulation and storage spanning 10s ka to Ma (Fig. 1) (*6*, *7*, *12*). Geophysical methods image shallow storage chambers and the upper parts of deeper source reservoirs at active volcanoes, with a few datasets imaging the entire crust (*13*–*15*). Interpretations of geophysical data suggest that magma throughout such systems has high average crystallinity (“mush”); large, low-crystallinity magma chambers that could supply *M* > 7 explosive eruptions have not been imaged (*12*).

Numerous previous studies have proposed that the size, frequency, and composition of large explosive eruptions are primarily controlled by processes in the shallow chamber (*2*, *4*, *5*, *16*). In these models, the chamber grows incrementally by intrusion of smaller magma batches sourced from one or more deeper reservoirs, eventually accumulating the volume required to supply a large eruption. Shallow storage timescales are interpreted to be short because they represent only the final magma batch intruded before eruption (*17*). Intruded magma may “rejuvenate” a silicic mush to create a low-crystallinity, eruptible magma (*18*–*20*). Eruption may be caused by internal mechanisms that produce overpressure, such as buoyancy or chamber inflation (*2*, *21*), or by external triggers, such as far-field stresses or foundering of the chamber roof (*22*–*25*). Models for eruption frequency and volume typically choose the magma composition and supply rate to the chamber and test the consequences for eruption (*2*, *4*, *5*, *16*, *21*).

Controls on the composition and supply rate of magma sourced from deeper reservoirs remain uncertain. Thermal models have investigated the conditions required to produce magma in the mid- to lower crust and store this magma at shallow depth (*26*–*28*). In these models, melt fraction is controlled only by temperature. Magma is assumed to leave the reservoir whenever the melt fraction is higher than a critical value, typically ~0.5 (*2*, *16*, *26*, *27*, *29*). Across this “critical melt fraction” (CMF; also termed the “solid-to-liquid transition”), the rheology of the magma transitions from that of a high-crystallinity mush hosting melt within the pore-space of a solid crystal framework, to that of a low-crystallinity melt hosting suspended crystals (*30*, *31*). The mechanism in these models by which the magma leaves the reservoir and transits the crust to the shallow chamber or erupt at surface is not specified, but transfer is usually assumed to be rapid (instantaneous), suggesting upward flow via dikes (*32*).

Such models omit key controls on magma accumulation in a source reservoir. First, they neglect melt fraction and composition change by segregation: the physical separation of melt and crystals (*33*, *34*). Segregation can occur by processes such as crystal settling at high melt fraction and reactive percolative flow and compaction at low melt fraction (*35*). Segregation must occur to drive chemical differentiation, causing changes in local melt fraction and composition that deviate substantially from purely thermal models (*35*). Second, they ignore controls on how magma leaves a source reservoir surrounded by cooler crust (*4*). This issue has been primarily addressed in the context of magma leaving a shallow chamber to erupt at surface, but similar considerations pertain to deeper source reservoirs, concerning the force(s) causing magma to leave the reservoir, controls on rock failure and fracture propagation, the thermal viability of magma-driven fractures (*4*), and magma transport involving ductile rather than brittle deformation, such as diapirism (*36*).

Here, we use numerical modeling to test the hypothesis that the size, frequency, and composition of large-scale explosive eruptions may be strongly controlled—or even dominated—by the coupled physical and chemical processes that accumulate magma in a deeper source reservoir and deliver this magma to the edifice. The source reservoir processes investigated here could also control the size, frequency, and composition of magma batches delivered to incrementally constructed plutons and batholiths (*37*, *38*).

### Source reservoir processes

In our numerical model, a source reservoir is formed by intrusion of parental magma originating from the deeper crust or upper mantle (*26*, *33*). The model captures the thermal response of the crust to magma intrusion, including phase change. However, unlike purely thermal models, it also captures melt and solid separation by compaction and reactive flow. Here, we use compaction in its most general sense to encompass all mechanisms by which melt fraction changes in response to gradients in solid (crystal) velocity, including (for example) sedimentation of crystals onto the base of a chamber (“sedimentation compaction”), rearrangement of crystal packing in a cumulate mush (“mechanical compaction”), and changes in crystal shape that allow deformation of the mush (“viscous compaction”) (*35*). The model solves numerically the equations describing transport of heat via conduction and advection in a reservoir created by repeated magma intrusions and mass and momentum transport via reactive flow of buoyant melt relative to the compacting crystal mush, following the approach in (*33*) (see Materials and Methods). Their model includes no mechanism for magma to leave the reservoir. Here, we go beyond previous approaches that assume magma can leave a reservoir if the melt fraction is higher than the CMF (*26*–*28*).

### Magma ascent to the edifice

Magma transfer between a source reservoir and a shallower chamber may occur via dykes (Fig. 1A) (*32*), diapirs (Fig. 1B) (*36*), or slow percolative flow of melt through a mush system that spans the crust (Fig. 1C) (*12*). Dyke transfer through cool, solid portions of crust is initiated by the upward propagation of magma-filled fractures. The key control on fracture propagation is not the tensile strength of the crust; rather, it is the rate of cooling and freezing of magma in the dyke (*4*). Magma flow must advect heat into the fracture more rapidly than it is lost by conduction to the surrounding rock to avoid “thermal death.” A minimum (critical) overpressure in the source reservoir is required to drive magma into the fracture at the required rate. If the fracture propagates to the shallow chamber, a feeder dyke can develop that allows rapid transfer of magma from source reservoir to chamber (*32*). Once the magma is transferred, flow ceases and the dyke will freeze. A new fracture must propagate from the source reservoir to transfer the next batch, which may exploit previous dykes or other weak zones but must again avoid thermal death.

**Fig. 2.**
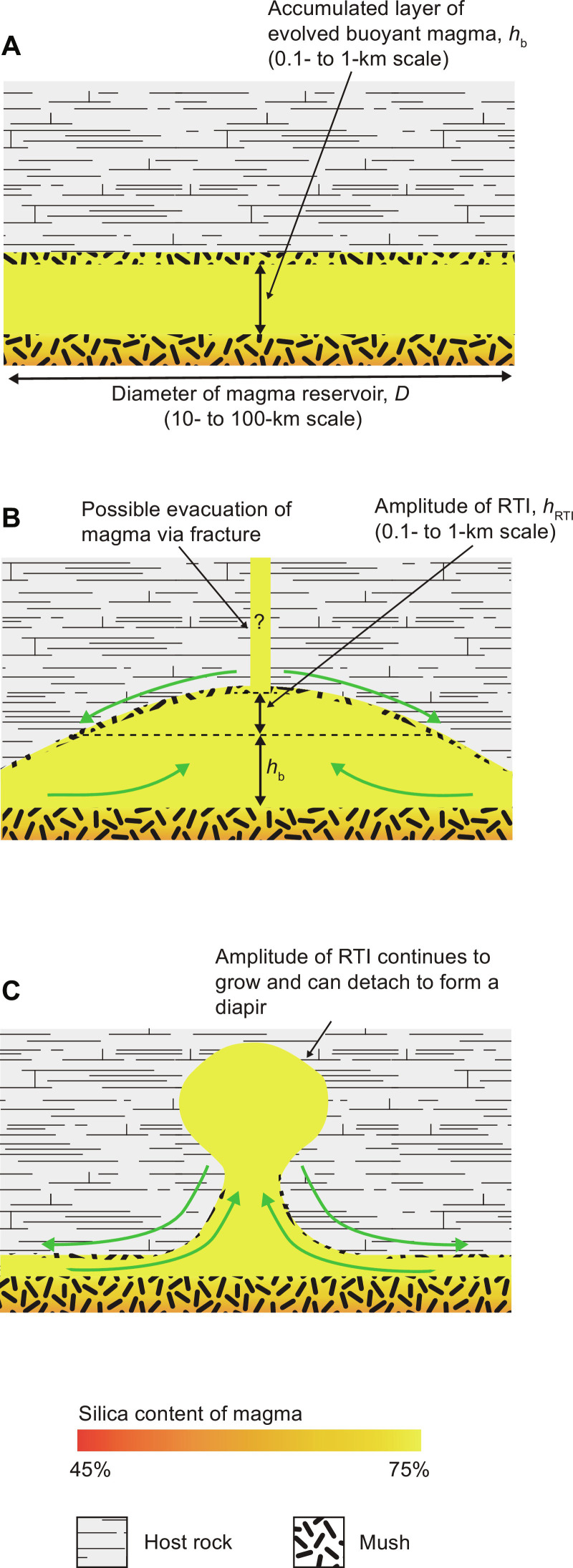
Development of an RTI in the source reservoir. (**A**) Percolative reactive flow leads to the formation of a layer of buoyant, low-crystallinity magma. (**B**) The buoyancy of the accumulated magma causes an RTI to develop as observed in analog experiments (*39*). The upwelling layer induces tensile stresses in the overlying crust, which can facilitate evacuation of the accumulated magma via buoyancy-driven fractures. (**C**) Continued growth of the RTI can lead to the detachment of a diapir. In our results, the combined buoyancy of the magma layer and RTI exceeds the critical buoyancy for fracture propagation in all cases, so the magma evacuates before a diapir can form. Note that the RTI amplitude (*h*_RTI_) and layer thickness (*h*_b_) are small compared to the lateral extent of the reservoir. Green arrows in (B) and (C) indicate flow of magma and displacement of crust during growth of the RTI.

Our numerical model results [see also (*33*)] show that the upward flow of buoyant melt through the source reservoir causes a layer of low-crystallinity, silicic magma to accumulate (Fig. 1D). Whenever such a buoyant layer is present, it will grow a Rayleigh-Taylor instability (RTI): The layer upwells into the overlying reservoir and crust, and magma drains laterally from the layer into the upwelling portion (Fig. 2B) (*37*). The upwelling grows in amplitude and can eventually detach to form a diapir (Fig. 2C). If this occurs, then the diapir migrates upward, but unlike dike transport, the migration rate is low, limited by the high viscosity of the surrounding crust rather than the comparatively lower viscosity of the magma (*36*). The diapir also loses heat to the surrounding cold crust as it ascends and may suffer thermal death before reaching the shallow chamber. If the magma in the layer develops a buoyancy overpressure during RTI growth that exceeds the critical overpressure required for fracture propagation to the shallow chamber, then the layer can drain rapidly via the resulting feeder dyke before detachment occurs.

Here, we capture the growth of the RTI using the approach in (*39*) (Fig. 2; see Materials and Methods). As melt accumulates, the magma layer thickness increases; as the RTI grows, its amplitude increases. Consequently, the total buoyancy of the magma layer increases. If the total magma buoyancy exceeds the critical overpressure, then a feeder dike is created which allows rapid magma ascent (*32*). We do not address the cause of dike arrest in the shallow chamber, as this has been investigated previously (*5*, *40*); rather, we assume an initial intrusion depth for the first batch of magma at 5 km, consistent with interpreted shallow magma storage in natural systems (*4*–*6*). Subsequent intrusions occur at a depth controlled by the local density contrast between magma and crust, which serves here as a proxy for rigidity contrasts resulting from changes in rock composition or melt fraction (see Materials and Methods). The critical overpressure does not correspond to the buoyancy required for magma to ascend to a specific depth; larger overpressure in our model does not lead to direct eruption of magma that evacuates the source reservoir.

The timescale of fracture propagation and magma transfer via the feeder dike is rapid compared to the timescale of magma accumulation, so in our model, we instantaneously transfer accumulated magma from the reservoir to the chamber as soon as the critical buoyancy is reached (see Materials and Methods). Only magma with a melt fraction higher than the CMF is transferred, so we account for both the overpressure and melt fraction required for magma to leave the reservoir. We term the process of magma leaving the reservoir and intruding the chamber a “magma evacuation.”

The failure to image low-crystallinity magma reservoirs in geophysical surveys, along with petrological and geochemical data consistent with magma storage at low melt fraction, has led to the development of conceptual models in which magma storage and chemical evolution occur primarily in high crystallinity mush reservoirs, within which melt transport occurs by percolative reactive flow through the pore-space between crystals (*12*, *33*, *41*). In this conceptual model, the mush reservoir can extend to the shallow chamber, which represents a transient, high melt fraction “cap” accumulating buoyant, upward-percolating melt. Magma delivery from the source reservoir occurs by persistent, pervasive melt flow rather than the delivery of discrete batches via dykes or diapirs. We find that the critical overpressure for fracture propagation is always achieved before the RTI is fully developed, so we do not address diapiric magma ascent. However, our model does allow partial melting and upward melt migration to create a mush reservoir that delivers melt directly to the chamber by percolative flow. Hence, we test here two mechanisms for magma delivery to the chamber: rapidly via dikes that transit solid crust between the source reservoir and the chamber (Fig. 1A) and slowly via persistent melt flow through a mush reservoir that extends to the base of the chamber (Fig. 1C).

### Shallow magma storage and eruption

We implement a simple model for eruption from the shallow chamber rather than attempting to directly address the cause (*2*, *4*, *5*, *16*, *21*). Our focus here is on magma delivery to the chamber from the deeper source reservoir. We test different residence times for magma in the chamber, to determine the potential for thermal feedback between shallow and deeper processes if magma erupts soon after it enters the chamber (1 ka) or is stored for longer periods (5 to 25 ka), thus retaining heat and mass in the shallow crust. Although simple, our approach allows for long duration crystal storage in the shallow chamber: Only magma above the CMF is erupted after the chosen post-intrusion residence time, so magma that remains in the chamber can continue to host and grow crystals which can be erupted after intrusion of a later batch of magma evacuated from the source reservoir.

## RESULTS

### Development of buoyancy overpressure at the top of a magma layer

**Fig. 3.**
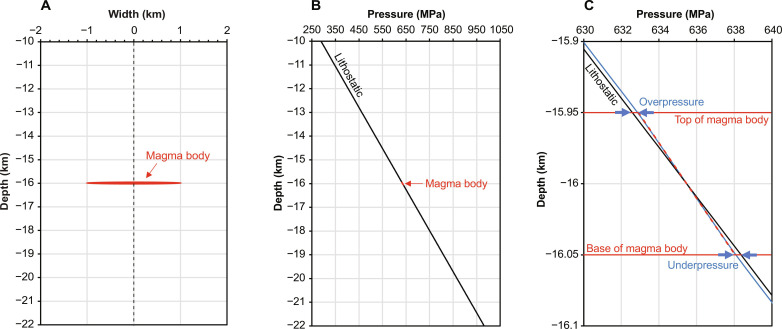
Simulated pressure around an elliptical magma body in viscous crust. (**A**) The body has a width of 2000 m, a thickness of 100 m, and a density of 2300 kg/m^3^ and is located at a depth of 16 km in crust with density 2950 kg/m^3^. Only a part of the 24-km-wide model domain is shown. The domain size was chosen to ensure that the model boundaries have no impact on the simulated pressure. (**B**) Pressure along a vertical profile through the center of the body. (**C**) Close-up of plot (B), focusing on the magma body. The curve labeled “lithostatic” represents the far-field pressure and the pressure before emplacement of the body; the dashed curve denotes the initial pressure within the magma body immediately after emplacement, and the blue curve denotes the pressure 10 ka after emplacement. The overpressure (with respect to lithostatic) at the top of the body and the underpressure at the base of the body are indicated. The pressure returns to lithostatic above and below the body.

Buoyancy has been invoked as a source of overpressure at the top of a confined layer or chamber containing magma with a lower density than the surrounding crust (*2*, *21*). However, Gregg *et al.* (*24*) argued that the lithostatic and magmatic pressures at the top of a chamber must be equal and suggested that the magma is therefore underpressured relative to the undisturbed lithostatic pressure. Here, we begin by calculating the pressure around an elliptical, buoyant magma body embedded in viscous crust, solving for incompressible Stokes flow using the Imperial College Finite Element Magma Reservoir Simulator (Fig. 3) (*42*, *43*).

We find that the crust and magma pressures at the top of the body are indeed equal, but only because the presence of the magma body causes a deviation of the pressure in the overlying crust from the undisturbed lithostatic pressure. The pressure at the top of the body is higher than lithostatic, and the pressure at the base is lower than lithostatic (Fig. 3C). The pressure at the depth of the center of mass of the body is lithostatic. We conclude that buoyancy does indeed induce an overpressure relative to lithostatic at the top of a confined layer or chamber, of order Δρ*gh*/2 where Δρ is the density contrast and *h* is the layer thickness [see also (*21*)]. This overpressure causes lateral flow of the crust to allow the RTI to grow and provides an overpressure that can drive fracture propagation into the extending crust above the upwelling layer (Figs. 1A and 2B; see Materials and Methods).

### Magma accumulation, ascent, and eruption in a typical example case

We report a specific example case in detail, before reporting summary findings for a wide range of cases to determine key controls on the volume, frequency, and composition of magma delivery from a source reservoir to a shallow chamber. The example case was chosen because values of the material properties lie in the mid-range of those expected (table S1), and analysis of numerous numerical simulations confirms that the same fundamental dynamics are observed across cases for different combinations of material property values. In our chosen example, 100-m-thick basalt sills are intruded to create and sustain a source reservoir, consistent with extensive data confirming that large explosive eruptions are the surface manifestation of magmatic systems created and maintained by the intrusion of mantle-derived basalt (*6*, *10*, *11*). The chosen sill thickness is consistent with previous thermal models (*26*).

The first sill is intruded at 20-km depth; later, sills are intruded around a depth that is controlled by the local density (see Materials and Methods). The initial intrusion depth is chosen in this example to match geophysical data that image a source reservoir beneath Yellowstone, extending from ~15- to 45-km depth (*13*). Similar reservoirs are imaged beneath other active volcanic systems (*14*, *15*, *44*). As we show later, our modeled source reservoir extends over a similar depth range. A total thickness of 15 km of basalt is emplaced at an average parent magma flux of 25 km^3^ ka^−1^, typical of large volcanic systems (*6*, *10*, *11*, *26*, *28*, *33*), into solid crust with an initial geotherm of 25°C km^−1^. The area of the magma reservoir is c. 5000 km^2^ and corresponds to the area of a circular reservoir with a diameter of 50 km, comparable with the Yellowstone caldera (*1*). Note that the crust thickens by less than 15 km because evolved magma is lost from the top of the magma system via eruptions. The remaining model parameters are typical of crustal magma reservoirs (table S1).

**Fig. 4.**
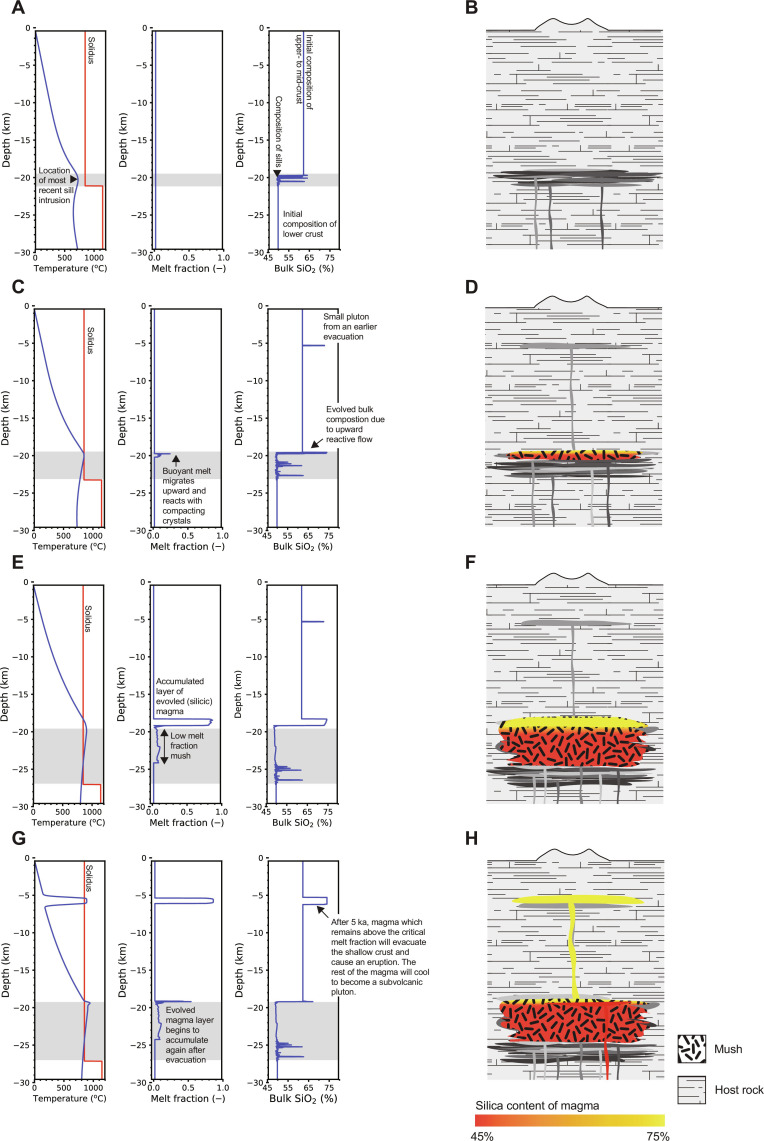
Simulated temperature, melt fraction, and composition as a function of depth, along with a schematic interpretation of the results. (**A** and **B**) Snapshot at 255 ka after the onset of parent magma intrusions, during the incubation phase; (**C** and **D**) 695 ka after the onset of parent magma intrusions, during the first growing phase; (**E** and **F**) 1479 ka after the onset of parent magma intrusions, during the first active phase, and (**G** and **H**) 1481 ka after the onset of parent magma intrusions, during the next growing phase after the first evacuation. Gray shaded box denotes the vertical extent of intruded parent magma at that time.

**Fig. 5.**
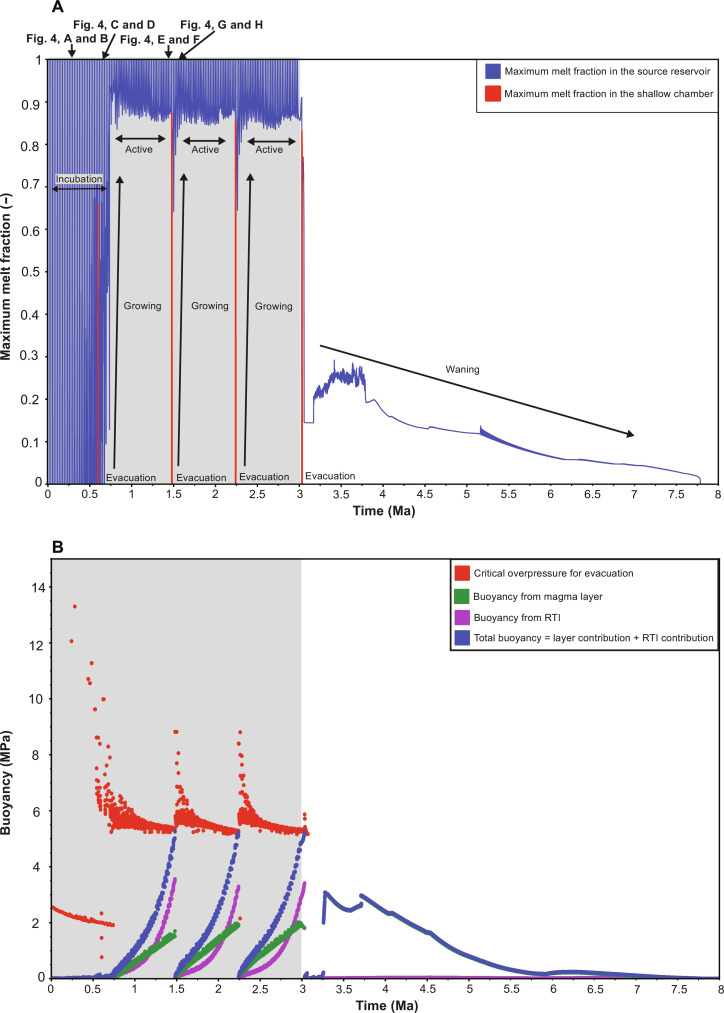
Time evolution of simulated melt fraction and magma buoyancy. (**A**) Maximum melt fraction in the source reservoir and shallow chamber; the time snapshots shown in Fig. 4 are indicated. After an initial incubation phase, during which the crust warms in response to sill intrusions (corresponding to each blue melt fraction spike), a persistent reservoir forms and melt accumulates by reactive percolative flow during the growing phase to produce a low-crystallinity, evolved layer of magma in the active phase. (**B**) Magma layer buoyancy and the critical buoyancy for evacuation. When the layer buoyancy exceeds the critical buoyancy, magma evacuates the source reservoir and intrudes the shallow chamber [red melt fraction spikes in (A)]. After a chosen shallow storage time (5 ka in this example), low-crystallinity (melt fraction higher than the CMF) magma present in the chamber can erupt. Ongoing percolative flow creates a new magma layer in the source reservoir after each evacuation, and the cycle repeats to create episodic evacuations. Gray shaded box denotes the period of parental magma intrusions into the source reservoir.

Results from our chosen example case are shown in Figs. 4 and 5 and movie S1. The reservoir initially enters the “incubation” phase (Fig. 4, A and B): The crust warms until melt is persistently present, and the reservoir enters the “growing” phase (Fig. 4, C and D). The incubation stage is also observed in thermal models [e.g., (*26*)]. However, our model predictions differ from those of thermal models because we capture the upward percolation of melt through the permeable mush reservoir that forms in response to ongoing sill intrusions (Figs. 1D and 4, C and D).

In our example case, a small silicic evacuation (of volume ~200 km^3^) occurs during the incubation phase (Fig. 5A), because a recent intrusion of parental magma thermally rejuvenates solidified silicic magma formed by upward percolation of melt following a previous sill intrusion. The local increase in temperature creates a small volume of silicic magma, which is surrounded by warm crust so only a small critical overpressure is needed for evacuation (Fig. 5B and see Materials and Methods). As we show later, evacuations driven by thermal rejuvenation are rare in our model results (see Discussion). All magma with a melt fraction greater than the CMF (0.6 in our example case; table S1) evacuates the reservoir and rapidly transits the crust to the subvolcanic chamber. Upon arrival, the magma cools to form a silicic pluton within ~0.8 ka, well within the chosen 5-ka shallow storage time (Fig. 4, C and D). We investigate in a later section the effect of varying the CMF and of longer and shorter duration shallow storage before eruption.

Once melt is persistently present in the source reservoir, it percolates upward during the growing phase and accumulates beneath the overlying solid crust. Eventually, a layer of low-crystallinity magma forms; this is the “active” phase (Figs. 4, E and F, and 5A) (*33*) during which the reservoir contains eruptible magma which has evolved (silicic) composition because it accumulates near the top of the reservoir where the temperature is low. At this time in our example case, the silicic magma layer is underlain by ~5 km of mush with melt fraction ~6%. The magma is buoyant with respect to the surrounding crust, so the layer begins to upwell as an RTI.

As melt continues to percolate upward through the mush, the magma layer grows in thickness; the RTI also grows in amplitude, until the total magma buoyancy exceeds the critical pressure for evacuation. All accumulated magma with melt fraction above the CMF then evacuates the reservoir and rapidly transits the crust to occupy the subvolcanic chamber (Fig. 4, G and H). Here, the magma resides for our chosen time of 5 ka before an eruption occurs; all magma with a melt fraction above the CMF leaves the chamber, and the remainder cools and crystallizes to form a silicic pluton (Fig. 4H).

After evacuation, the source reservoir reverts to the growing phase, comprising a low melt fraction, non-eruptible mush. Buoyant melt percolates upward and accumulates until the reservoir again enters the active phase (Fig. 5A). As before, once the buoyancy exceeds the critical pressure, the accumulated magma evacuates the reservoir, transits the crust, and recharges the subvolcanic chamber, leading to another large eruption. This cycle repeats, giving rise to episodic evacuations with a much lower frequency than the parent magma intrusions into the source reservoir (Fig. 5). Each evacuation is followed by an eruption of magma remaining in the chamber after the chosen shallow storage time.

Once sill intrusions cease, the source reservoir enters the “waning” stage, during which it cools, although magma batches may continue to be supplied to the chamber until the reservoir has solidified. In our example case, the source reservoir delivers four batches of silicic magma to the subvolcanic chamber, yielding three corresponding large eruptions of ~2000 km^3^ separated by ~775 ka, comparable to the composition, volume, and frequency of observed, large magnitude, explosive silicic volcanism (*1*, *2*, *6*, *7*, *16*).

After the final evacuation and as it enters the waning stage, the source reservoir extends from 16- to 32-km depth and has low average melt fraction of ~10%, similar to source reservoirs imaged beneath Yellowstone (see movie S1) and other active volcanic systems (*13*–*15*, *44*). Melt is present in the slowly cooling reservoir for a further 4.5 Ma after the final evacuation and eruption (Fig. 5A); cooling is slow because the temperature in the reservoir is buffered at the solidus so is almost uniform. Conductive heat loss therefore occurs primarily at the top and base of the reservoir (movie S1). In contrast, the subvolcanic chamber never leaves the incubation phase because, although large in volume, magma evacuations from the source region are too infrequent and magma is lost from the chamber in large eruptions. Melt is present only transiently in the shallow chamber following each evacuation (Fig. 5A).

### Source reservoir controls on episodic magma delivery to the shallow chamber

Analysis of numerous simulation cases shows that partial melting of the crust above the source reservoir allows melt from the reservoir to percolate upward, so the top of reservoir becomes shallower with time; the base of the shallow chamber also moves downward as magma accumulates (Fig. 4). However, we see no cases where the top of the source reservoir meets the base of the chamber to allow percolative melt flow directly from the reservoir into the chamber (Fig. 1C). Consequently, magma delivery to the shallow chamber always occurs in distinct magma batches, transported via dykes created when the magma layer buoyancy in the source reservoir exceeds the critical buoyancy required to propagate a fracture to the chamber (Fig. 1A).

**Fig. 6.**
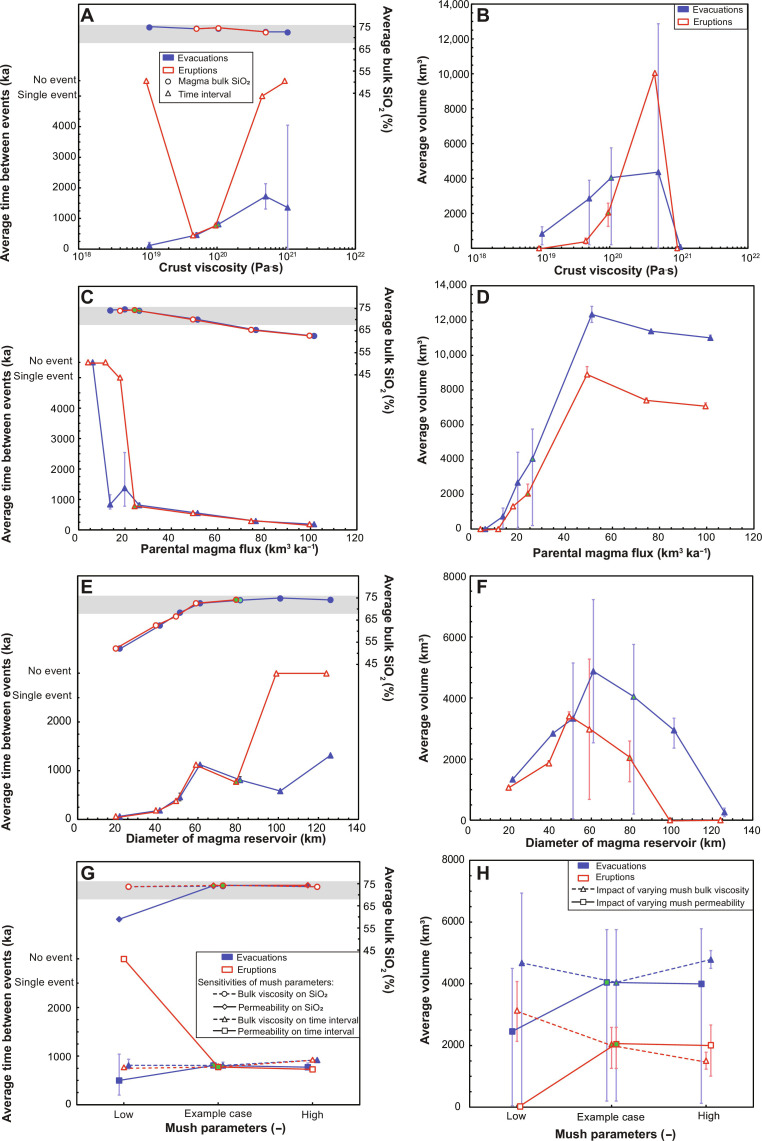
Impact of varying key uncertain model properties on the frequency, volume, and composition of magma evacuations from the source reservoir. Plots show the impact of varying (**A** and **B**) crust viscosity, (**C** and **D**) parental magma flux, (**E** and **F**) reservoir size, and (**G** and **H**) reservoir mush properties. The chosen shallow storage time is 5 ka. Error bars for a particular ordinate axis value show the minimum and maximum simulated outcomes over the range of property values explored in the other plots. In each plot, our chosen example case is shown in green. In plots (E) and (F), the mush bulk viscosity is varied over the range 10^14^ to 10^16^ Pa·s (the example case is 10^15^ Pa·s), and the mush permeability scaling factor is varied over the range 10^−9^ to 10^−7^ m^2^ (the example case is 6.05 × 10^−8^ m^2^; see table S1). Also shown are the corresponding data for eruptions.

We find that key controls on the temperature and composition of evacuated magma, the volume of evacuated magma, and the frequency (period) of evacuations are the rheology of the overlying crust, the reservoir diameter (size), and the rate of silicic magma accumulation in the reservoir by percolative melt flow (Fig. 6). The latter depends on the mush reservoir properties, and we show here the effect of varying three key uncertain model parameters: the mush permeability and bulk viscosity (*33*) and the flux of parental magma which controls the reservoir growth rate (*26*, *33*). The values of these key parameters control evacuations via a complex interplay of nonlinear processes within the source reservoir. Sensitivities to further uncertain properties are reported in a later section but do not affect the key findings.

The evacuated magma is generally cool and silicic (SiO_2_ > 68%), except for high parent magma fluxes, small reservoirs, and/or low rates of melt percolation and accumulation in the reservoir (Fig. 6, C and E to G). The composition of the magma is controlled by its temperature (fig. S1E); cooler magmas are more evolved and vice versa. High parent magma fluxes yield less silicic evacuations (Fig. 6) because each parent magma intrusion adds heat to the reservoir; high parent magma fluxes therefore correspond to high heat fluxes into the reservoir, resulting in higher reservoir temperatures. Similarly, small reservoirs have higher temperatures for a given parent magma flux; the accumulated magma is therefore warmer and less evolved in both cases (Fig. 6E). Smaller reservoirs also evacuate less evolved magma because they exhibit more rapid layer growth, reaching the critical buoyancy before there has been substantial differentiation. Source reservoirs with low mush permeability evacuate smaller volumes of less evolved magma (Fig. 6, G and H) because upward melt percolation is slower. Consequently, melt accumulates more slowly and deeper in the reservoir, where the temperature is higher.

The predicted time between evacuations varies over 10s - 1000s ka, and the predicted volume of evacuations over 10s to 1000s km^3^ (Fig. 6). Larger and less frequent evacuations are predicted from reservoirs overlain by crust with high shear viscosity (Fig. 6, A and B), because the RTI, and therefore buoyancy, grows more slowly, allowing a thicker layer of magma to accumulate before evacuation. Crust viscosity is correlated to temperature and rock composition (*45*): Larger evacuations are likely sourced from reservoirs overlain by cooler, stronger crust. However, if the viscosity is too high, then RTI growth is almost entirely suppressed so the magma layer may never accumulate sufficient buoyancy for a large evacuation. Evacuations in models with high crust viscosity are very small and occur only due to thermal rejuvenation or during the waning phase when a small magma layer is overlain by dense mafic crust, so the layer buoyancy is unusually high (see Materials and Methods).

Larger and more frequent evacuations are predicted from reservoirs created by high parent magma flux (Fig. 6, C and D), because the reservoir is warmer and therefore has higher average melt fraction, allowing more rapid regrowth of the eruptible layer. High magma fluxes likely originate from melting of anomalously hot or wet mantle (*6*, *10*–*12*, *26*). However, if the flux is too high, then the crust overlying the reservoir becomes warmer, so the critical buoyancy decreases (see Materials and Methods), yielding smaller evacuations.

Evacuation volume and frequency are also controlled by the magma reservoir size (diameter), with evacuation volume, and the time between evacuations, both reaching a maximum at an optimum reservoir size (Fig. 6, E and F). The RTI grows more rapidly in larger reservoirs, but the layer thickness grows more slowly for a given parent magma flux, because the injected heat is distributed over a larger area, so the reservoir is colder. The interplay of these two competing effects yields an optimum reservoir size for the largest and least frequent evacuations. The predicted optimum reservoir size is consistent with the largest observed volcanic systems (*6*, *13*, *14*). Very large volcanic systems must be sustained by exceptionally high parental magma fluxes, otherwise the source reservoir remains too cold to produce large evacuations that can feed large eruptions. Overall, the largest, and least frequent, evacuations are predicted from large reservoirs that are cooler and therefore deliver cool, silicic magma, consistent with the composition of observed large, explosive eruptions (*1*, *4*, *6*, *7*, *46*).

### Impact of shallow magma storage timescale

**Fig. 7.**
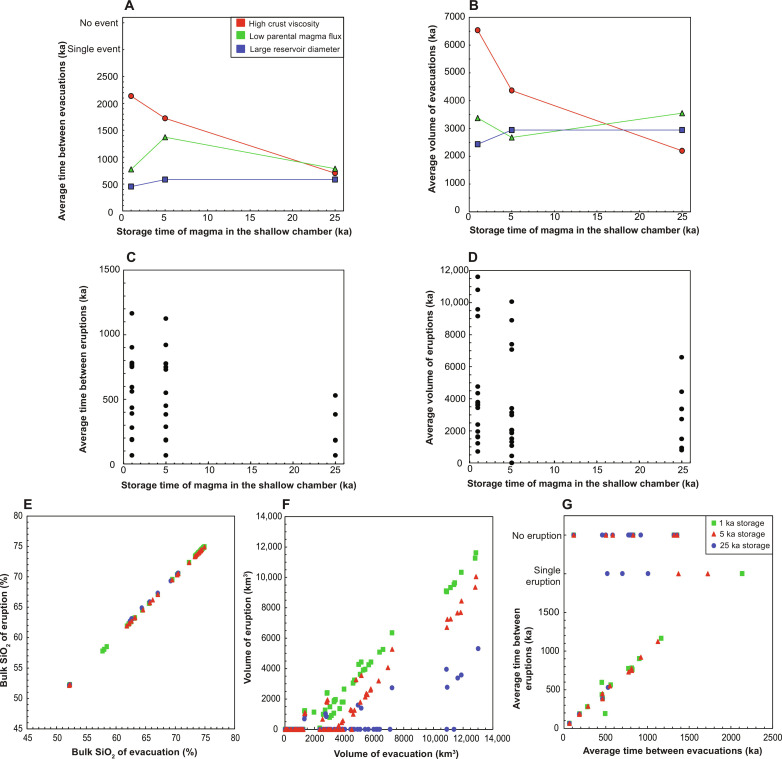
Impact of varying shallow magma storage time on predicted model behavior. Plots show the impact on the average frequency and volume of (**A** and **B**) evacuations and (**C** and **D**) eruptions and on the correlation between evacuation and eruption (**E**) composition, (**F**) volume, and (**G**) time interval. [(A) and (B)] Plots show the impact of shallow storage time on evacuations from large reservoirs (*D* = 100 km), reservoirs supplied by low parental magma fluxes (18.85 km^3^ ka^−1^), and reservoirs overlain by viscous crust (5 × 10^20^ Pa·s) because these show the greatest sensitivity to storage time. In all cases not shown here, evacuations are independent of shallow storage time (see fig. S8 for the full set of results). Plots (C) and (D) show the impact of varying storage time on eruptions for all evacuation parameters shown in Fig. 6. [(E) to (G)] Plots show (E) evacuation and eruption composition, (F) volume, and (G) time interval, respectively, for different storage times.

Shallow storage time has little impact on the volume, frequency, or composition of magma evacuations from the source reservoir over the range of 1 to 25 ka tested (see fig. S8), because there is no significant thermal feedback between the chamber and reservoir. In most cases, the source reservoir is too deep for conductive heat transfer from the chamber to substantially affect temperature within or above the reservoir. Only if the source reservoir is particularly cool (corresponding, as shown in the previous section, to large reservoirs supplied by low magma fluxes), or the overlying crust has particularly high viscosity, do we observe weak relationships between shallow storage time and magma evacuation: frequency increases (Fig. 7A) and volume decreases (Fig. 7B), because the crust above the source reservoir is comparably warmer, so the critical buoyancy is comparatively lower, allowing evacuations of more rapidly assembled magma volumes that may be smaller.

In contrast, predicted eruption volumes are strongly affected by shallow storage time, with eruptions becoming smaller (Fig. 7D) with increasing storage time, irrespective of the source reservoir behavior. Eruptions become smaller because magma in the chamber cools and crystallizes during storage, so smaller volumes remain with melt fraction above the CMF. Eruptions become less frequent, because an increasing number of evacuations yield no eruption, but the recorded time interval between eruptions decreases (Fig. 7C) because many modeled systems produce no eruptions or just one eruption over the model timescale (Fig. 7, F and G). Eruptions are always smaller than evacuations (Fig. 6, B, D, F, and H); if evacuations are small, then there may be no corresponding eruption, because the magma cools and crystallizes within the chamber before it erupts. Eruption frequency is, therefore, either the same as, or smaller than, evacuation frequency (Fig. 6, A, C, E, and G), and eruptions become less frequent with increasing shallow storage time. Note that in our model, evacuated and erupted magma compositions are identical (Fig. 7E) because we do not include processes that could give rise to further differentiation in the shallow chamber during storage.

### Parent magma composition

Our canonical example assumes that the source reservoir is created by intrusion of basalt sills. However, in many systems, the reservoir is created by intrusion of intermediate magma (*6*, *12*, *18*–*20*, *47*). Here, we confirm that the same fundamental dynamics are observed irrespective of the parental magma composition. We keep all model parameters the same as in our chosen example case (table S1) but intrude sills containing intermediate magma.

The results are qualitatively similar to those obtained in the example case (Figs. 4 and 5). As before, the reservoir passes through the incubation and growing phases, before entering the active phase (see fig. S6 and movie S2). Once the critical buoyancy is reached, magma evacuates the reservoir to supply an eruption. However, the high melt fraction, evolved magma layer accumulates more rapidly when the parent magma is initially more evolved. Consequently, there are larger and more frequent eruptions compared to our chosen example case. Nonetheless, the same overall behavior is observed: the episodic creation of large volumes of eruptible silicic magma and its delivery to the edifice.

### Sensitivity analysis on model parameters

Several parameters described in the model formulation may play a role in controlling evacuation volume and frequency but were not tested in Fig. 6 or the previous sections. Jackson *et al.* (*33*) reported a comprehensive sensitivity analysis of the parameters that control melt accumulation in crustal mush reservoirs, and the results of this informed the parameters tested in Fig. 6.

**Fig. 8.**
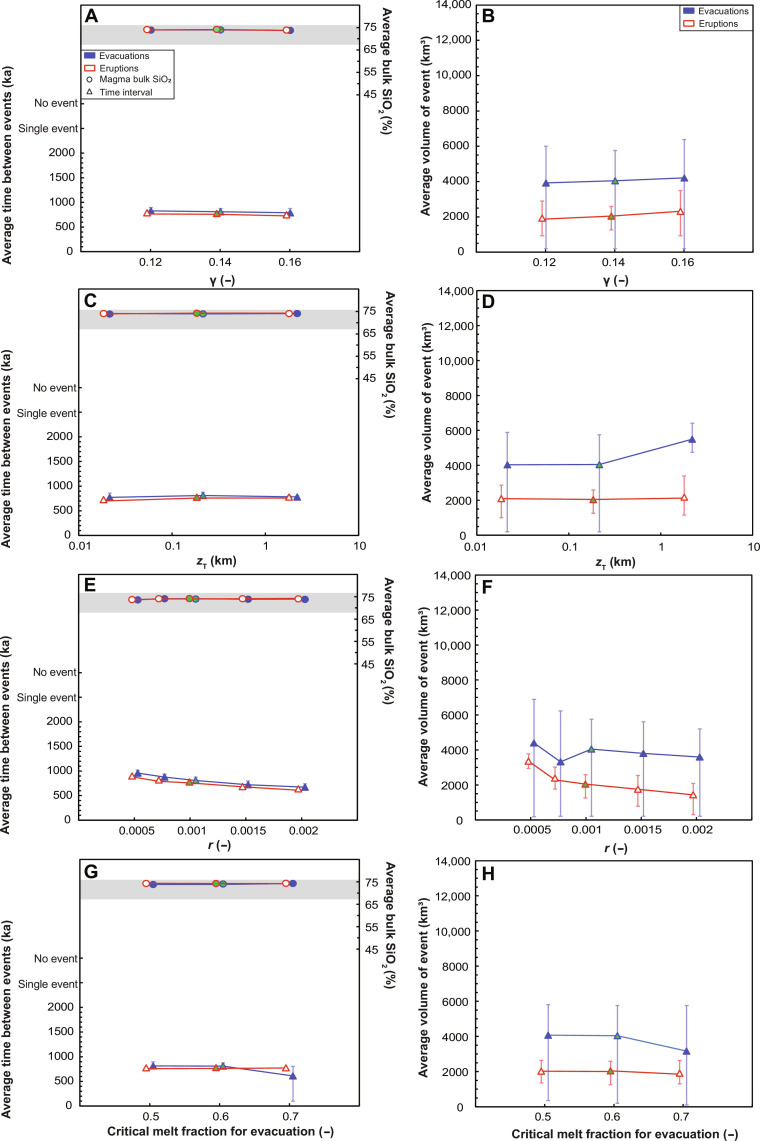
Sensitivity of the predicted evacuation volume, frequency, and composition to parameters related to evacuation. (**A** and **B**) Ratio of pressure at the tip of the dike to pressure in the magma reservoir (γ); (**C** and **D**) distance over which the temperature gradient is recorded in Eq. 10 (*z*_T_); (**E** and **F**) the initial amplitude of the RTI (*r*); and (**G** and **H**) the CMF. Error bars for a particular ordinate axis value show the minimum and maximum simulated outcomes over the range of property values explored in Fig. 6. Also shown are the corresponding data for eruptions. The example case is highlighted in green.

We focus in this sensitivity analysis on model parameters that control magma evacuation and eruption, including the vertical interval (*z*_T_) above the reservoir over which we calculate the temperature gradient used to predict the critical overpressure, the dimensionless cooling parameter (γ) used to predict the critical overpressure, the CMF above which magma can leave the reservoir or erupt from the chamber, and a scaling factor (*r*) for the initial RTI perturbation *h*_RTI_O__ which we relate to the magma reservoir diameter *D* by

$$h_{{\text{RTI}}_{O}}=\mathrm{rD}$$ (1)

(see Materials and Methods) (Fig. 8). In all cases, the parameters were varied over ranges that are reasonable for geological systems. Other parameters in each sensitivity test correspond to the values used in our example case (table S1 and Figs. 4 and 5).

We find that most of these model parameters have little or no impact on evacuation or eruption volume, frequency, or composition, giving confidence that our key findings are not conditional to a specific suite of model parameters. Varying *r* has a small effect on evacuation volume and frequency: As *r* increases, evacuations become smaller and more frequent, because the RTI grows more rapidly for a larger initial layer topography *h*_RTI_O__ (Fig. 8, E and F; see Materials and Methods). However, the range of *r* values tested (table S1) corresponds to initial layer topography over the range of 12.5 to 50 m, yet the evacuation frequency varies by only 30% and the evacuation volume by 19%. The largest value of *z*_T_ tested yields larger average evacuation volume (Fig. 8D), because there are no small evacuations caused by thermal rejuvenation during the incubation phase; later, large evacuations that lead to eruptions are not affected. The largest CMF tested also yields slightly smaller and more frequent evacuations (Fig. 8, G and H). We discuss the role of the CMF in a later section.

## DISCUSSION

### Deep versus shallow accumulation and storage of silicic magma

Our numerical model results suggest that the accumulation of sufficient volumes of silicic magma to supply a large, explosive eruption occurs in the deeper source reservoir. The magma accumulates by reactive percolative melt flow; this process also causes chemical differentiation of the parent basalt or intermediate magma. Differentiation and accumulation are therefore closely related. The accumulated magma remains trapped in the reservoir until large volumes are present, because of the high overpressure required for a silicic magma-driven fracture to successfully propagate upward through cold crust. The overpressure required for fracture propagation scales with magma viscosity and temperature gradient (see eq. S10 in Supplementary Methods): A cool, high viscosity silicic magma requires an overpressure that is four to five times higher to leave the reservoir as compared to a hot, low viscosity basaltic magma. Models that assume silicic magma evacuation can occur as soon as the melt fraction exceeds a CMF neglect this important limitation on magma transport through cold crust.

**Fig. 9.**
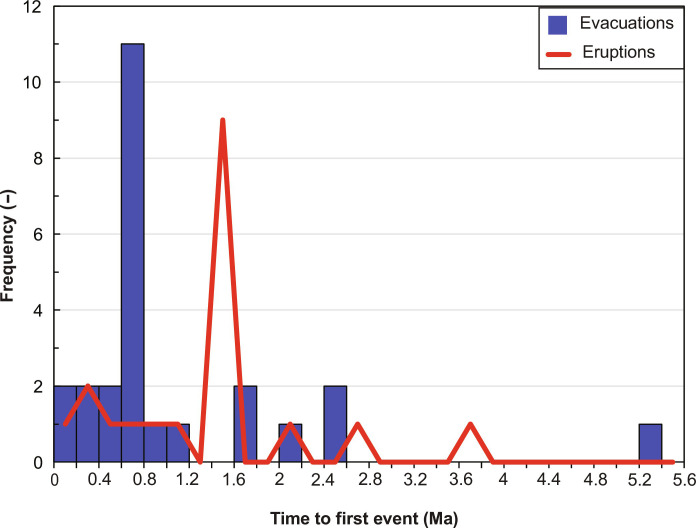
Time from the onset of parental magma intrusions to the first evacuation and eruption of magma. Results from all parameters tested in Fig. 6 are summarized. The chosen shallow storage time is 5 ka.

Following initiation of the magmatic system, defined here as the first intrusion of parent magma into the crust, melt segregation and accumulation in the source reservoir produce the first magma evacuation over timescales ranging up to 5 Ma but more typically of order 100’s ka (Fig. 9). The timescale from initiation to first evacuation depends on the parent magma intrusion rate and size of reservoir but is typically too short for thermal conduction of heat from the reservoir into the upper crust to cause substantial warming. Silicic magma evacuations are therefore emplaced into relatively cold upper crust, so they cool rapidly (timescales of order 0.1 to 10 ka). The temperature of the evacuated magma is also low, which further reduces the longevity of shallow storage of eruptible magma.

Thermal priming of the upper crust to allow long-term, shallow magma storage requires much longer timescales of deeper magmatism—of order 3 to 4 Ma—before the onset of shallow magma emplacement (*28*). Deep magmatism must be long duration to allow the slow process of thermal conduction to transport heat upward over kilometer-scales to the shallow crust. Such long duration magmatism must be sustained by intrusion of large volumes of parent magma which remains in the mid- to lower-crust and supplies heat, but not magma, to shallower depth. Karakas *et al.* (*28*) imposed this condition in their model by externally specifying the timing of magma evacuation rather than allowing it to emerge in response to melt segregation processes in the reservoir.

Our results suggest that a dynamically evolving magma reservoir produces evolved, eruptible magma over much shorter timescales, although the reservoir overall may be equally long-lived. Accumulation at depth, with transient storage in a subvolcanic chamber, is thermally favorable. In our model, the magma is transferred in a single batch via a single dyke, but multiple dykes could result in multiple transfers of smaller batches over a short timescale, resulting in rapid assembly of magma in the chamber (*6*, *7*).

### Magma evacuation and thermal rejuvenation

Rapid heating has been proposed as a mechanism for melt fraction increase in crustal mush reservoirs leading to magma mobilization and eruption, in a process termed “thermal rejuvenation” or “defrosting” (*18*–*20*). In this model, a cool or cold mush reservoir at low melt fraction is heated by intrusion of new, hot magma, causing the melt fraction in the surrounding mush to increase. Thermal rejuvenation is widely invoked as a mechanism to create low-crystallinity magmas in mush reservoirs, often over short timescales (*7*, *18*–*20*).

In our results, evacuation of low-crystallinity magma primarily occurs in response to the accumulation of buoyant magma via percolative melt flow and is decoupled from the intrusion of new parent magma; >80% of evacuations in Fig. 6 correspond to this mechanism (see fig. S1E). However, we also observe two additional evacuation triggers. The first, and least frequently observed (<6%), corresponds to thermal rejuvenation: A sill is intruded below solidified silicic magma, which is heated and remelted such that the melt fraction exceeds the CMF. The resulting small magma volume can evacuate only if the overlying crust is hot, so there is an unusually low critical overpressure for evacuation. These evacuations are too small to drive large eruptions.

The second cause is an increase in magma layer buoyancy in response to intrusion of parental magma into the base of an evolved magma layer that is just below the critical buoyancy. We term this “buoyancy rejuvenation.” Evacuation occurs because of the increase in buoyancy rather than an increase in melt fraction. Buoyancy rejuvenation is again rare in our models (<12%), and the reservoir needs to have been primed by melt flow and accumulation beforehand. However, buoyancy rejuvenation can create large evacuations, so can be a cause of large eruptions. We suggest that buoyancy rejuvenation is a hitherto unrecognized cause of large eruptions.

### Magma evacuation and the CMF

The insensitivity of evacuation behavior to the CMF is a key finding (Fig. 8, G and H). It is common in conceptual and numerical models to assume that magma becomes mobile and evacuates its host reservoir once the CMF is reached (*2*, *16*, *26*, *27*, *29*). Here, we show that buoyancy, rather than the CMF, is the key control on evacuation from source reservoirs. The buoyancy required for evacuation is only reached once a high melt fraction, evolved magma layer has formed in response to percolative melt flow. Because the accumulated magma is already at high melt fraction, the value of the CMF plays no substantial role in controlling evacuation volume, frequency, or composition over the broad range of values tested.

### Relationship between magma evacuation and eruption

Our model is focused on source reservoir controls on magma delivery to a subvolcanic chamber. Conditions specific to a given system that are not considered here, such as local tectonic stress, the presence of preexisting faults and other zones of weakness, and the exsolution of volatiles, likely control the style and exact timing of eruption from the subvolcanic chamber after recharge (*2*, *6*, *16*, *22*–*25*, *48*–*50*). Our model does not capture these controls on eruption or processes that lead to further differentiation in the shallow chamber. However, our results show that the magma volume available to erupt decreases with increasing duration of shallow storage (Fig. 7, C and D), suggesting that shallow storage for the largest eruptions is transient after chamber recharge, which may itself trigger eruption: Rapid emplacement of very large volumes of silicic magma can create overpressure, reactivate preexisting faults or other zones of weakness or induce caldera collapse (*2*, *6*, *22*, *23*).

**Fig. 10.**
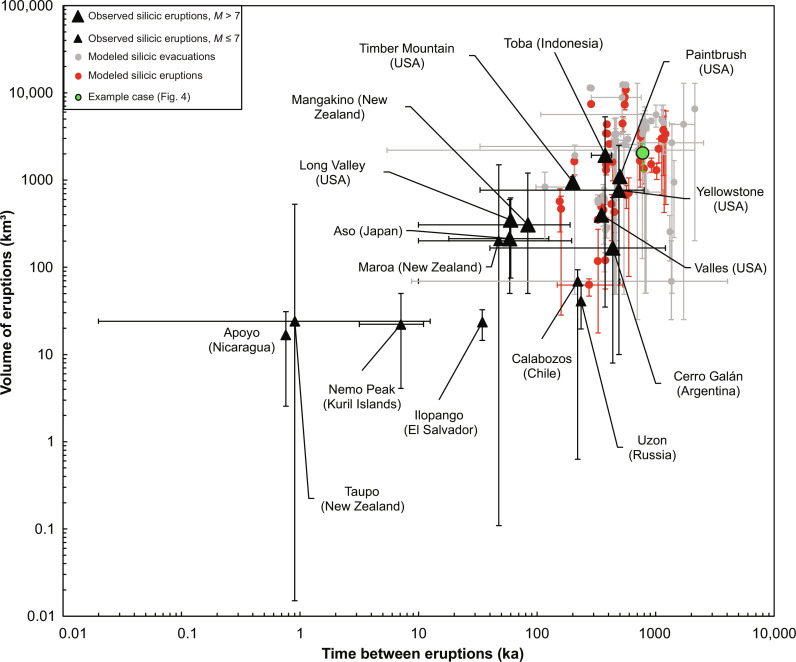
Comparison between observed and modeled eruption frequency and volume. Black triangles represent observed silicic eruptions from caldera volcanoes (see table S2). Circles represent model results with multiple silicic evacuations (gray) and eruptions (red). Error bars represent the minimum and maximum volume and frequency. Our chosen example case, shown in Fig. 4, is highlighted.

The composition, temperature, volume, and frequency of magma evacuations from the source reservoir predicted by our model are consistent with those observed in large explosive eruptions (Fig. 10). We suggest that magma accumulation in long-lived mush reservoirs and its buoyancy-controlled release dictate the overall size, frequency, and composition of these eruptions, by controlling the episodic creation of large volumes of eruptible silicic magma and its delivery to the edifice. Shallow storage is transient (of order a to 10’s ka) compared to the interval between eruptions (or order 100’s ka), so the size, frequency, and composition of evacuations and eruptions are closely correlated (Fig. 7, E to G). Longer duration shallow storage reduces the volume and number of eruptions (Fig. 7, E and F), favoring pluton formation over eruption.

A key question is why small eruptions are observed between caldera-forming events. We hypothesize that these are sourced from residual magma or mush remaining within the subvolcanic chamber; the same processes of reactive percolative flow and compaction as in the source reservoir could produce low-crystallinity magma in the shallow chamber; models of these processes likely need to be extended to include a free volatile phase, which is why they are omitted here. Alternatively, small eruptions could be induced as precursor events when a large batch of new magma begins to enter the chamber before the next caldera-forming eruption (*6*, *7*, *51*). In some cases, parent magma could bypass the source reservoir and directly intrude the shallow chamber; controls on when this would occur remain poorly understood (*40*).

Our results suggest that the largest and least frequent eruptions originate by rapid segregation of melt from large mush reservoirs sustained by high parent magma fluxes and overlain by strong crust. The erupted magma is silicic because large source reservoirs are comparatively cool. There is an optimal reservoir size to deliver the largest eruptions. Geophysical imaging can be used to determine the depth and size of these source reservoirs but has so far imaged only the thick, persistent, low melt fraction mush predicted by our modeling (*13*–*15*) rather than the transient, evolved magma layer that is evacuated to feed an eruption. No large shallow chambers or deeper layers occupied by low-crystallinity magma have been detected. This may be because of the restricted spatial resolution of geophysical data at source reservoir depths, because geophysical data have not been acquired in a system where a large magma layer is now accumulating, or because magma accumulation occurs in some different location. Our model results suggest that magma layers in a deep source reservoir are of order 1 to 2 km thick before evacuation. Imaging at higher spatial resolution, possibly through use of joint inversion techniques, may reveal such bodies.

## MATERIALS AND METHODS

### Source reservoir model formulation

The numerical model used to describe the storage, differentiation, and accumulation of magma in the source reservoir is reported in (*33*) so only a brief summary is presented here. The model includes the repeated intrusion of sills into the crust to create and sustain the reservoir, the associated transport of heat via conduction and advection, and mass transport via reactive, percolative flow of buoyant, evolved melt through a compacting, crystalline mush. We do not include a free volatile phase in the source reservoir model, as we expect volatiles to remain dissolved at the high confining pressures encountered at depth (*52*).

The model solves numerically the conservation equations for heat, mass, and component transport including thermal diffusion but neglecting component diffusion and the Darcy equation for conservation of momentum with viscous compaction of the crystalline matrix. The equations are solved in one dimension (1D) using a finite-difference method (*33*, *53*). Several 2D and 3D numerical magma reservoir models that include similar physics have been reported recently, but the computational cost of such models is too high for them to be applied at the vertical and lateral scales considered here; moreover, none yet include the repeated intrusion of sills to create and grow the reservoir (*54*–*56*). We therefore consider a 1D model, to develop a first-order understanding of magma storage, differentiation, and accumulation in a source reservoir and the impact of these processes on the delivery of magma to a volcanic edifice. We use a 2D model for the less computationally expensive calculation of the pressure within and around a buoyant magma body in the crust (Fig. 3).

Transport of components by the melt changes the local bulk composition which also modifies the local melt fraction. A simple two-component, eutectic phase diagram is used to capture the impact of the local bulk composition on the melting behavior. The phase diagram is adjusted to match the experimentally determined melting behavior of intruding basalt and host crust (fig. S1A) (*11*, *57*, *58*). The SiO_2_ content of the melt and solid phases is related to composition using the same experimental melting data (fig. S1B). Solid density and melt density and viscosity are also composition dependent (fig. S1, C and D) (*21*, *59*–*71*). Our numerical modeling results show that the composition of magma that accumulates in a source reservoir is primarily controlled by magma temperature (fig. S1E).

Key mush properties controlling the rate of percolative melt flow and associated compaction of the crystalline matrix are the permeability of the mush and the effective bulk viscosity of the mush. Consistent with (*33*, *52*) (and numerous references therein), we relate permeability to melt fraction (porosity) using an equation of the form

$$k=k_{c}\varphi^{3}$$ (2)

where φ is the porosity and *k*_c_ is the characteristic mush permeability, which depends on mush grain size *d* according to (*33*, *53*)

$$k_{c}=bd^{2}$$ (3)

The constant *b* is adjusted to match experimental data. For typical crustal mush reservoirs, *k*_c_ varies over the range 1 × 10^−9^ to 1 × 10^−7^ m^2^ (table S1).

The mush effective bulk viscosity is also related to melt fraction using an equation of the form (*33*, *53*, *72*, *73*)

$$\eta =\eta_{0}\varphi^{-0.5}$$ (4)

where η_0_ is a characteristic mush shear viscosity, which is estimated to vary over the range 1 × 10^14^ to 1 × 10^16^ Pa·s (table S1). We demonstrate the impact of these uncertainties on model predictions in Fig. 6. Equations 2 and 3 are appropriate for porous media flow in mush reservoirs at low melt fraction, when the melt occupies the pore-space between the grains. Equation 4 provides a simple model for the dependence of effective mush bulk viscosity on melt fraction when the solid crystals form a contiguous framework. We discuss these equations further and application of the model at higher melt fraction in a later section.

Sill intrusions are modeled by adding new nodes (grid cells) with the properties of the intruding magma (temperature, composition, and derived properties such as viscosity and density) and shifting downward the existing nodes (see movie S1). We assume therefore that parental magma intrusion is accompanied by downward movement of the underlying crust, consistent with previous models of repetitive sill intrusion (*26*, *27*, *33*). Sills typically intrude in our model into solid crust or mush at low melt fraction, so we assume that rapid sill intrusion is facilitated by brittle failure of the surrounding (partially molten) rock. We neglect mechanical work done by the intruding magma on the surrounding rock, consistent with the evidence that such work is limited to a narrow region of order a few meters adjacent to the contact even when a dyke or sill intrudes into low melt fraction mush (*74*–*76*).

In the canonical example shown here, 100-m-thick basalt sills are initially intruded into the crust around a target depth of 20 km and then around a target depth that is controlled by the density contrast between the intruding sill and the mush. The density contrast is used as a proxy for rigidity contrasts and rheology anisotropy resulting from changes in rock composition or mush melt fraction, not because buoyancy directly controls intrusion depth [see (*33*) for details]. Sills are intruded at a randomly selected depth within 300 m above and below the target depth (*33*).

Initially, the crust has a geotherm of 25°C km^−1^ and an intermediate composition to 20-km depth, with a dry, refractory composition below this. The entire crust is initially below the solidus *T*_s_ so there is no melt present. The refractory deep crust has a higher solidus (*27*).

The rate at which parental magma sills are intruded is dependent on the chosen volumetric flux of the basalt (*q*_h_) into the magma reservoir and the diameter (*D*) of the axisymmetric cylindrical geometry assumed for the sills. The time interval between intrusions (*T*_I_) is given by

$$T_{I}=\frac{0.1\pi D^{2}}{4q_{b}}$$ (5)

In our example case, the chosen flux of 25 km^5^ ka^−1^ and a diameter of 80 km means that sills are intruded at a rate of one sill per 20 ka.

Upward percolative flow causes evolved melt to accumulate at the top of the reservoir to form a layer of low-crystallinity, evolved (silicic) magma (Fig. 1D) which can be evacuated from the layer and ascend through the crust to intrude a subvolcanic chamber. Sills intruding the shallow crust, transporting magma from the source reservoir, are initially intruded at a target depth of 5 km and then around a target depth that is again controlled by the density contrast between the intruding sill and the surrounding crust. We find in our model that early evacuations cool to form low-density, silicic plutons that typically act as barriers to the upward propagation of later magma-filled fractures, preventing direct eruption to surface irrespective of the buoyancy overpressure in the source reservoir. Natural controls on shallow intrusion depth include local contrasts in rheology due to variations in lithology or density or the presence of local structures such as faults, local deviatoric stresses, and volatile exsolution (*5*, *40*) [see also (*33*) for a discussion].

### Application of the source reservoir model at high melt fraction

As discussed in (*33*), the numerical model used here to describe the storage, differentiation, and accumulation of magma in the source reservoir assumes mass transport via reactive, percolative flow of buoyant melt through a mush comprising a contiguous framework of crystals that deforms via viscous creep. However, the model is applied throughout the domain, regardless of local melt fraction. Estimates of the melt fraction at which a crystal framework forms vary widely (over the range of 0.4 to 0.7), and the value of this CMF likely depends on whether melt fraction is locally increasing or decreasing the local shear stresses and strain rates and the crystal morphology and size distribution (*30*, *31*, *55*, *77*–*80*). Melt fractions higher than this estimated range are present in each sill immediately after intrusion and in the silicic magma layers that form in response to compaction and reactive flow. However, consistent with (*33*), we argue that the formulation captures enough of the physics to yield informative results.

Keller and Suckale (*81*) presented a continuum formulation to model magma reservoirs that spans the high and low melt fraction domains. Following their approach, we consider melt-solid separation, for which the separation velocity in our 1D model can be described by an equation of the form

$$v_{s}=C∆\rho g$$ (6)

where ∆ρ is the melt-crystal density contrast and *g* is the acceleration due to gravity. At low melt fraction, the coefficient *C* is chosen such that Eq. 6 corresponds to Darcy’s Law, in which case

CD=kμm (7)

where *k* is the permeability given by Eq. 2 (*81*). At high melt fraction, the coefficient *C* should be chosen such that (*6*) corresponds to hindered settling (*81*)

$$C_{\text{HS}}=\frac{2d^{2}\left(1-\varphi \right)\varphi^{5}}{9\mu_{m}}$$ (8)

Comparing μ_m_*C*_D_/*d*^2^ and μ_m_*C*_HS_/*d*^2^ to remove the common factors of grain size and melt viscosity (fig. S2), it is apparent that, for our chosen material properties, values are reasonably similar at high melt fraction: *C*_HS_ is typically higher than *C*_D_, but the maximum difference is a factor of approximately 4 over the melt fraction range of 0.6 to 0.7 and is much smaller than the two orders of magnitude uncertainty range in permeability that is tested in the models (table S1). Thus, our Darcy-based formulation reasonably captures melt-solid separation at high and low melt fraction.

We next consider the effective mush bulk viscosity. Numerous studies have suggested that both the bulk and shear viscosities of a compacting mush increase with decreasing melt fraction (porosity) below the CMF, although the form of the relationship depends on the deformation mechanism(s) and remains an area of active research (*31*, *33*, *53*, *72*, *73*, *81*–*83*). Many models have assumed a relationship of the form η = η_0_/φ where η_0_ is the shear viscosity (cf. Eq. 4). Above the CMF, the effective shear and bulk viscosities are dominated by the properties of the melt rather than the properties of the crystalline matrix (*81*).

Figure S3A shows our simple model for the melt fraction dependence of the mush bulk viscosity (Eq. 4), compared against a model based on (*31*, *80*, *81*). A common feature of published models is that bulk and shear viscosity of the mush decrease rapidly as the melt fraction approaches the CMF, assumed in fig. S3A to have a value of 0.5. In contrast, our simple model maintains a large bulk viscosity to high melt fraction. This viscosity offers a resistance to compaction [defined generally to mean crystals moving closer together such that the crystal fraction increases and melt fraction decreases; see (*35*)] that is apparently inconsistent with the dynamics of a suspension of crystals in melt.

Published models of mush bulk (and shear) viscosity are appropriate for 2D and 3D models of crystal-melt dynamics, which capture bulk flow of melt and crystals driven by convection, as well as the relative motion of melt and crystals associated with compaction (*54*–*56*). Direct application of these viscosity models in a 1D model of magma dynamics, such as the one used here, predicts rapid differentiation in a single sill to form a layer of highly evolved magma above a refractory residue (fig. S3B). Isolated sill intrusions with such extreme compositional differences are rarely observed; rather, differentiation is observed over much longer (kilometers) length scales, consistent with our model predictions (*33*, *64*). In 2D and 3D models, such layers in a single sill are mixed and homogenized by convection (*54*–*56*, *84*–*86*).

The simple model of mush bulk viscosity used here captures, to first order, the increase in viscosity with decreasing melt fraction observed in previous studies of mush rheology at low melt fraction but maintains a large viscosity at high melt fraction to suppress rapid, short length-scale crystal-melt separation in individual sill intrusions which is caused by convective mixing. We test the impact of uncertainty in the modeled value of characteristic mush viscosity over a two orders of magnitude range (table S1).

High melt fractions are present in the intruding sills over very short timescales (of the order of hundreds of years) because the sills cool very rapidly, losing heat to the surrounding reservoir and/or crust. Irrespective of our simple approach to modeling magma dynamics, the sill cooling timescale is correct, because the rate of heat loss from each sill is dominated by conduction (*34*). In a single sill, the model captures crystal-melt separation to yield relatively subtle differentiation, with a slightly more evolved top and a more refractory base (fig. S3B), consistent with observations of isolated sill intrusions (*53*, *87*–*89*). This differentiation occurs by compaction and percolative reactive melt flow after the sill has cooled to form a mush. Ongoing compaction and reactive flow in response to repeated sill intrusions eventually yield reservoir-scale differentiation.

High melt fractions are persistently present in silicic magma layers until the magma leaves the reservoir. However, the rate of growth of the layer is controlled by the rate of delivery of new melt by reactive flow and compaction of the underlying mush, where the model formulation is valid. Thus, we argue that the model captures the (re)growth rate of the layers, which is shown to be a key control on the delivery of magma to a volcanic edifice to drive a large-scale eruption.

### Evacuation of magma due to buoyancy

A major limitation of the reservoir model reported in (*33*) is that it omits any mechanism for magma to leave the reservoir and migrate through the crust to intrude a shallow, subvolcanic chamber or erupt to the surface. Accumulation of evolved melt at the top of the reservoir by compaction and reactive flow creates a magma layer that is buoyant relative to the overlying and surrounding crust. Whenever such a buoyant layer is present, it will grow an RTI. The combined buoyancy arising from the layer thickness and the upwelling RTI causes magma to flow into fractures opening in the overlying crust at the top of the RTI (Fig. 1). Previous work has shown that such magma-filled fractures will propagate upward and reach the subvolcanic chamber if the total layer buoyancy exceeds a critical value (*4*)

12Δρg(hb+hRTI)>σT (9)

where *h*_b_ is the buoyant magma layer thickness, *h*_RTI_ is the RTI amplitude, Δρ is the average density contrast between the magma and surrounding crust, *g* is the gravitational acceleration, and σ_T_ is the critical buoyancy (*4*)

$$\sigma_{T}={\left[\frac{2c_{p}E^{2}}{\gamma L}\;{\left(\frac{3\kappa \mu_{m}}{\pi }\right)}^{\frac{1}{2}}\left|\frac{\mathrm{dT}}{dz_{T}}\right|\right]}^{\frac{2}{5}}$$ (10)

In Eq. 10, *c*_p_, κ, μ_m_, and *L* are the specific heat capacity, thermal diffusivity, shear viscosity, and latent heat of the magma entering the dike, dTdzT and *E* are the temperature gradient and elastic modulus in the overlying crust, and γ is a dimensionless “freezing parameter.” The RTI amplitude grows as (*39*)

hRTI=hRTI0etcτe (11)

where *h*_RTI_0__is the initial amplitude of the instability, *t*_c_ is the growth time, and

$$\tau_{e}=\frac{6\pi \mu_{c}}{\mathrm{Dg}∆\rho }$$ (12)

where μ_c_ is the shear viscosity of the overlying crust, and *D* is the diameter of the RTI, which here we equate to the reservoir diameter.

The propagating magma-filled fracture creates a dike that provides a conduit for magma to transit from the source reservoir to the chamber consistent with numerous field observations and previous models (*6*, *32*, *37*, *38*, *90*, *91*). However, magma will only be able to migrate out of the reservoir via this conduit if it is sufficiently melt-rich (*77*–*79*). Here, consistent with previous studies (*2*, *16*, *26*, *27*, *29*), we assume that buoyant magma leaves the reservoir and is transported via the conduit only if it has a melt fraction higher the CMF (taken to be 0.6 in the example cases shown in Figs. 4 to 7; see table S1). As we show, model predictions are insensitive to the chosen CMF over the likely range of 0.5 to 0.7 (Fig. 8, G and H).

The buoyancy driving evacuation has two sources: the buoyancy caused by the presence of the confined magma layer at the top of the reservoirs (Fig. 3) and the buoyancy caused by growth of an RTI in the layer (Eq. 11; Fig. 2). We address these two sources of buoyancy in the next two sections, and then the model was used to determine the critical buoyancy for evacuation.

### Buoyancy from magma layer thickness

We assume that the magma layer is laterally confined (Fig. 2) so is buoyant relative to the surrounding crust. The buoyant layer thickness (*h*_b_) includes all vertically connected magma that is buoyant from the top of the reservoir down. In the numerical model, *h*_b_ is calculated as the connected group of nodes from the top of the reservoir which have a melt fraction φ > 0 and are buoyant relative to the overlying crust. The density of the layer is calculated as an average of the connected nodes and used to calculate the density contrast Δρ between the buoyant magma layer and the surrounding crust.

### Buoyancy from RTI

Rayleigh-Taylor instabilities develop naturally whenever buoyant magma layers form. We are interested in estimating the buoyancy created by an RTI and its influence on the evacuation of magma from the reservoir. Our analysis is based on the earlier work of Bremond d’Ars *et al*. (*92*) and Seropian *et al*. (*39*) which build on the fundamental theory of Whitehead and Luther (*93*) for a thin buoyant layer beneath a much thicker and much more viscous (by many orders of magnitude) layer. Bremond d’Ars *et al*. (*92*) developed the theory to include a growing layer rather than one of fixed thickness. Seropian *et al*. (*39*) considered the geologically relevant situation where the layer is laterally confined with width less than the fastest growing wavelength for an unconfined layer. Both studies report experimental results which agree with the predictions of the theory for a wide range of viscosity ratios.

From this body of work, we conclude that the growth of an RTI is characterized by three stages: an initial stage of unconfined growth, an intermediate stage of confined growth, and a final stage of detachment. We find that the critical overpressure for fracture propagation is always achieved before the RTI is fully developed, so the third stage is not reached. Thus, our focus is on the first two stages.

The initial stage of unconfined RTI growth begins when a buoyant magma layer forms near the top of the reservoir. Initially, the instability has a very small wavelength, amplitude, and growth rate. As the magma layer increases in thickness, the fastest-growing wavelength of the RTI increases, which increases the amplitude of the RTI. The wavelength of the RTI is given by

$$\lambda =\frac{4\pi }{2.88}\dot{h_{b}}t_{u}{\left(\frac{\mu_{c}}{\mu_{m}}\right)}^{1/3}$$ (13)

where $\dot{h_{b}}$ is the growth rate of the buoyant magma layer, and *t*_u_ the time elapsed since layer formation. These parameters are both extracted from the numerical model. We assume that the diameter of the magma layer cannot exceed the diameter of the reservoir; consequently, when the wavelength of the RTI reaches the magma reservoir diameter (= *D*), the RTI becomes confined and the unconfined growth stage ends.

On the basis of the experimental observation and theory (*39*, *92*, *93*), we adopt an exponential growth law for the amplitude *h*_RTI_ of the confined instability (Eqs. 11 and 12), where *t*_c_ is the time since the onset of the confined instability (fig. S4). Numerical experiments not reported here show that the timescale of unconfined growth is short compared to timescale of confined growth (*t*_u_ << *t*_c_). Therefore, to reduce model complexity, we neglect the unconfined growth stage in the results reported here. Instead, we simply assume that confined growth of the RTI begins as soon as a buoyant magma layer has formed. Failure of the overlying crust occurs during the exponential growth of the RTI.

Our exponential growth model is dependent on the initial amplitude of the instability, *h*_RTI_O__ (Eq. 11). The value of the initial amplitude is arbitrary, in that the initial magma layer will not be perfectly flat; rather, there will be some topography at the contact between the accumulating magma layer and the overlying roof rocks. In our model, the initial instability is given by Eq. 1, with *r* = 0.001 in our canonical example, yielding plausible geologically controlled topography of order 10’s m for the range of magma reservoir diameters (*D* = 20 to 125 km) tested. The timescale for growth (Eq. 11) is only weakly dependent on the choice of the initial amplitude so long as *h*_RTI_O__ ≪ *h*_RTI_, which is valid for cases of geological interest at long time scales. As we have shown, model results are insensitive to *r* over a geologically reasonable range (Fig. 8).

### Critical buoyancy for evacuation

For the magma in the melt-rich layer to migrate upward out of the magma reservoir into a subvolcanic chamber, the buoyancy needs to be large enough to propagate a magma-filled fracture to the subvolcanic chamber. We term the buoyancy required for this the critical buoyancy, and the criterion we use here is based on the models developed in (*4*, *94*) which show that magma-driven fracture propagation is primarily controlled by the balance of heat addition and loss from the magma rather than the strength of the crust.

The fracture must propagate fast enough that the magma does not freeze during ascent, which requires that the fracture widens more rapidly due to magma addition than it narrows due to freezing. Rubin (*94*) showed that this criterion for the thermal survival of magma-driven propagating fractures can be expressed in terms of a dimensionless parameter, γ, given by

γ=2(3κμmπσT)12cpL|dTdzT|(σTE)−2 (14)

which describes the ratio of the pressure at the tip of the fracture (caused by low pressure from magmatic volatiles or host rock pore fluids) to the pressure in the magma reservoir. Assuming that magma within the dike has a constant average viscosity, Rubin (*94*) found that dikes cannot propagate unless γ < 0.12 to 0.16. Jellinek and DePaolo (*4*) rearranged Eq. 14 to determine the buoyancy necessary to successfully propagate a magma-filled fracture, which we equate with the critical buoyancy (Eq. 10). In our canonical example, we assumed the median value of γ = 0.14.

The critical buoyancy for magma evacuation changes dynamically throughout the lifetime of the magma reservoir to reflect the viscosity of the evolved magma and the temperature gradient in the overlying crust. We discuss the development of buoyancy and magma evacuation further in the next section. Here, the temperature gradient is calculated over the first *z*_T_ = 200 m of crust above the magma reservoir, as this generally represents the largest gradient that the magma will experience on its path to the subvolcanic chamber. As we have shown, model results are only weakly dependent on γ and *z*_T_ (Fig. 8).

### Development of buoyancy and magma evacuation

The critical buoyancy for evacuation and the total buoyancy in the magma reservoir both vary throughout the life of the reservoir. The time evolution of the critical and total buoyancy for our canonical example case, plus the respective contributions to the total buoyancy from the magma layer and associated RTI, are shown in Fig. 5B.

Initially, during the incubation phase (Fig. 5A), buoyant magma is only present immediately after a basalt sill intrusion. The magma volume is small, and there is insufficient time for an RTI to develop, so the total buoyancy in the magma reservoir is much lower than the critical buoyancy required for evacuation.

From 300 ka, as the magma reservoir enters the growing and then active phases (Fig. 5A), the critical buoyancy for evacuation initially increases, due to the presence of eruptible silicic magma which has a higher viscosity compared to the basalt magma that is intruded (Eqs. 9 and 10; fig. S1D). As the magma reservoir progresses through the growing and active phases, the critical buoyancy varies over two timescales: a shorter timescale related to individual sill intrusions and a longer timescale related to the thermal evolution of the mush reservoir.

Immediately after each basalt sill intrusion, the critical buoyancy decreases because the intruded basalt delivers a pulse of less evolved, less viscous melt via percolative flow into the overlying silicic magma layer, thus reducing the average viscosity of the magma in the layer, which in turn decreases the critical buoyancy (Eq. 10). As the melt composition evolves during ongoing percolative melt flow, the magma in the layer becomes more evolved and therefore more viscous, which increases the critical buoyancy. This process causes the oscillations in critical buoyancy observed after each sill intrusion (Fig. 5B).

The slow decline in critical buoyancy until magma evacuation reflects gradual warming of the overlying crust in response to the continued intrusion of basalt, thus decreasing the temperature gradient above the reservoir, thus reducing the critical buoyancy (Eq. 10).

During the growing and active phases, there is a large increase in the buoyancy of the magma in the reservoir; RTIs develop rapidly and eventually contribute about half of the total buoyancy. When the total buoyancy reaches the critical buoyancy, the melt-rich part of the magma layer is evacuated. The critical buoyancy then increases rapidly in response to the steeper temperature gradient above the reservoir as colder crust collapses onto hotter mush. At the same time, the total buoyancy decreases in response to the loss of magma. However, as the reservoir returns to the growing and then active phases (Fig. 5), the buoyant magma layer rebuilds, and the total buoyancy increases leading to another evacuation event.

In our canonical example, basalt sill intrusions stop at 3 Ma; however, a melt-rich magma layer is still present and growing due to the upward flow of buoyant melt through the slowly cooling mush, so a further eruption takes place during the waning phase, as observed in Fig. 5A. Buoyancy continues to fluctuate in the magma reservoir as the magma reservoir cools, even if the melt-rich magma layer is no longer present, due to buoyant mush forming as a result of ongoing percolative melt flow in the reservoir.

The proportion of the total buoyancy contributed by the RTI varies depending on the RTI growth rate. An RTI grows faster for larger magma reservoir diameter and smaller crust shear viscosity (Eqs. 11 and 12) thus contributing more to the total buoyancy. This is highlighted in fig. S5 (A and B), which show cases with low and high crust shear viscosities, respectively. In fig. S5A, low crust shear viscosity allows rapid RTI growth which dominates the total buoyancy, resulting in a larger number of smaller eruptions. Conversely, in fig. S5B, high crust viscosity yields slow RTI growth, allowing a thick, melt-rich magma layer to grow before the total buoyancy reaches the critical buoyancy, resulting in a small number of large eruptions.

If the flux of basalt magma into the reservoir and/or the rate of melt segregation within the reservoir is too low, then the magma reservoir may never produce an eruption because a buoyant magma layer is not present for long enough to allow an RTI to develop, or the layer remains too thin to produce sufficient buoyancy (e.g., fig. S5D). We observe similar time evolution of buoyancy irrespective of the composition of intruded magma (fig. S6).

### Magma evacuation and ascent

Magma evacuation via the dike(s) created by fracture propagation is initiated as soon as the buoyancy in the magma reservoir is greater than or equal to the critical buoyancy (Eq. 9). The melt-rich fraction of the buoyant magma is transported upward via dikes and intruded into a transient, subvolcanic chamber.

The volumetric flow rate of magma through a dike, *Q*, is given by (*95*)

$$Q=\frac{\Delta \rho gw^{3}L_{d}}{12\mu_{m}}$$ (15)

where *w* is the dike width and *L*_d_ is the horizontal breadth. For our canonical example, Eq. 15 predicts that all magma is evacuated from the reservoir to the subvolcanic chamber within a few months to years of failure of the overlying crust (fig. S7). The timescale of magma transit to the subvolcanic chamber is rapid compared to the thermal and chemical evolution of the magma reservoir. We therefore assume that magma transfer into the chamber occurs within a single time step in the model.

Magma evacuation, ascent, and emplacement into the subvolcanic chamber are modeled numerically by removing the nodes within the buoyant magma layer which have a melt fraction greater than or equal to the CMF and shifting down the overlying nodes to fill the gap thus created. We assume therefore that magma evacuation is accompanied by downward movement of the overlying crust. As the magma migrates through the dike and enters the subvolcanic chamber, it will effectively homogenize by convective mixing (*84*, *86*), so we calculate the average properties of the evacuated magma and assign these to the nodes that represent the magma. These nodes and their associated properties are then added to the model at the subvolcanic chamber depth, to represent intrusion of the evacuated and homogenized magma. During the user-defined shallow residence time (1 to 10s ka), we model cooling of the magma in the chamber. Magma which remains above CMF after the chosen residence time then erupts: Nodes representing this magma are removed from the model and recorded to determine the volume and composition of the eruption. The residence time and CMF do not substantially affect the source reservoir dynamics (fig. S8 and Fig. 8).

### Data on natural eruptions

Figure 10 shows observed data from volcanoes which have produced multiple, silicic, caldera-forming eruptions with average bulk dense rock equivalent (DRE) volume > 10 km^3^ to be classed as a large-scale eruption. The data sources are listed in table S2. The plotted triangles represent the average bulk DRE volume and average time between eruptions for a particular volcano. The error bars represent the minimum and maximum estimated average erupted volume and frequency.

For the modeled data, only model cases that erupted silicic magma were included. None of the sensitivity analysis cases reported in Fig. 8 are included in Fig. 10, as they do not vary substantially from the example case presented.

## Supplementary Materials

### This PDF file includes:

Figs. S1 to S8 Tables S1 and S2 Legends for movies S1 and S2 References
