# Supplementary material for: Source reservoir controls on the size, frequency, and composition of large-scale volcanic eruptions
Source: Sci Adv. 2024 May 10;10(19):eadd1595. doi: 10.1126/sciadv.add1595 (PMC11086626; doi:10.1126/sciadv.add1595)
Supplement: Supplementary file 1 — Figs. S1 to S8 Tables S1 and S2 Legends for movies S1 and S2 References [file sciadv.add1595_sm.pdf]

Science Advances
AAAS

Supplementary Materials for

Source reservoir controls on the size, frequency, and composition of large-scale volcanic eruptions

Catherine A. Booth *et al.*

Corresponding author: Catherine A. Booth, c.booth17@imperial.ac.uk;
Matthew D. Jackson, m.d.jackson@imperial.ac.uk

Sci. Adv. **10**, eadd1595 (2024)
DOI: 10.1126/sciadv.add1595

The PDF file includes:

Figs. S1 to S8
Tables S1 and S2
Legends for movies S1 and S2
References

Other Supplementary Material for this manuscript includes the following:

Movies S1 and S2

SUPPLEMENTARY MATERIALS

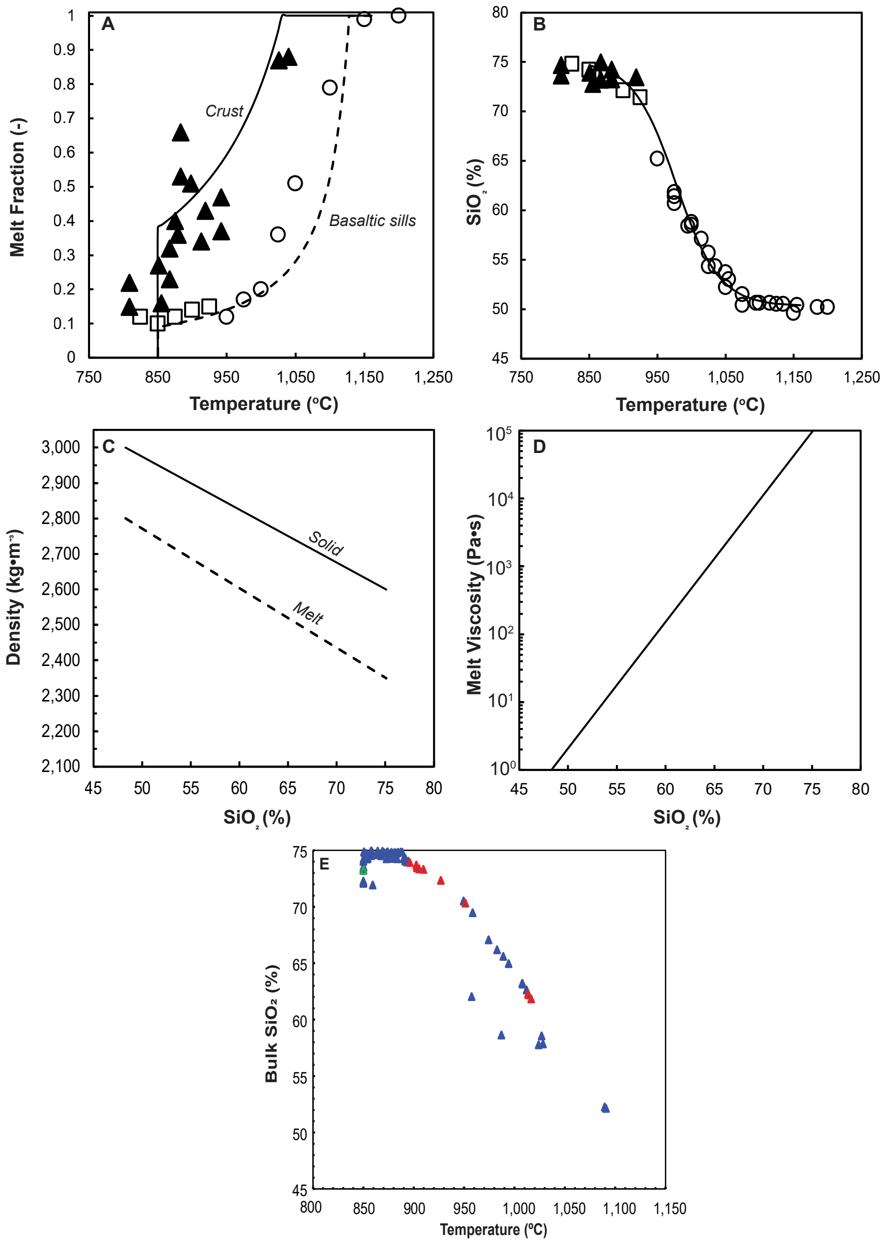

Figure. S1. Melting behavior, silica content, solid and melt density and melt viscosity. The modeled magma system phase equilibria and associated physical properties represent a relatively wet system. A. Melt fraction versus temperature for the modeled basalt and crust. Also shown are experimental data over the pressure range 400-900 MPa for basalt (circles and squares) and metagreywacke (triangles): triangles from *57*, circles from *58* and squares from *11*. **B.** SiO_2 content versus temperature. The experimental data are the same as in (A). **C** Solid and melt density versus SiO_2 content, modeled using data from *21, 59-65*. **D**. Melt viscosity versus SiO2 content, modeled using data from *66-71*. **E**. Temperature and bulk SiO_2 of magma evacuated from the source reservoir for the cases shown in Fig. 6. Green denotes evacuations caused by the thermal rejuvenation; red denotes evacuations caused by buoyancy rejuvenation; the remainder are caused by magma accumulation in response to reactive percolative melt flow.

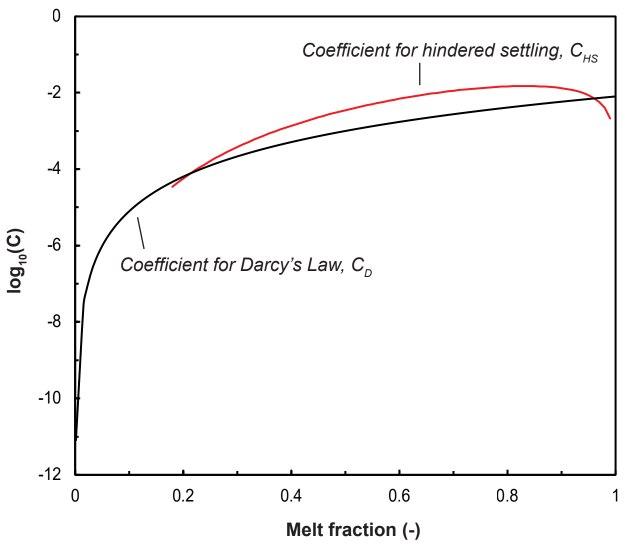

Figure S2. Comparison of coefficients used to calculate the melt-crystal separation velocity (eqn. 6) assuming hindered settling (C_{HS}) at high melt fraction and Darcy flow (C_D) at low melt fraction. We assume here that the Darcy coefficient can be used across the entire melt fraction range.

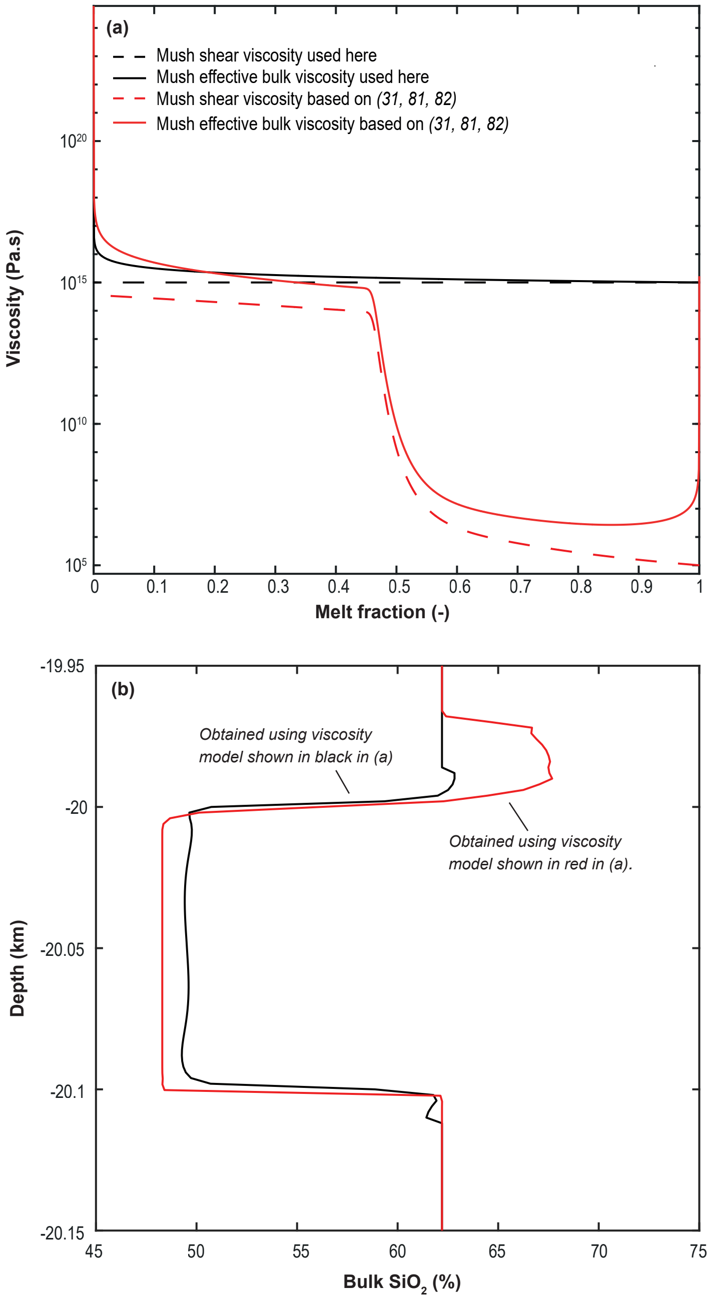

Figure S3. Comparison of different models for the melt-fraction dependence on mush bulk and shear viscosity. A. Effective mush shear viscosity (dashed lines) and bulk viscosity (solid lines) used here (black lines) compared to a model based on *31, 81, 82* (red lines), assuming a CMF of 0.5. **B.** Modeled silica content after solidification of a single 100m sill intruded at 20 km obtained using the viscosity models shown in (A).

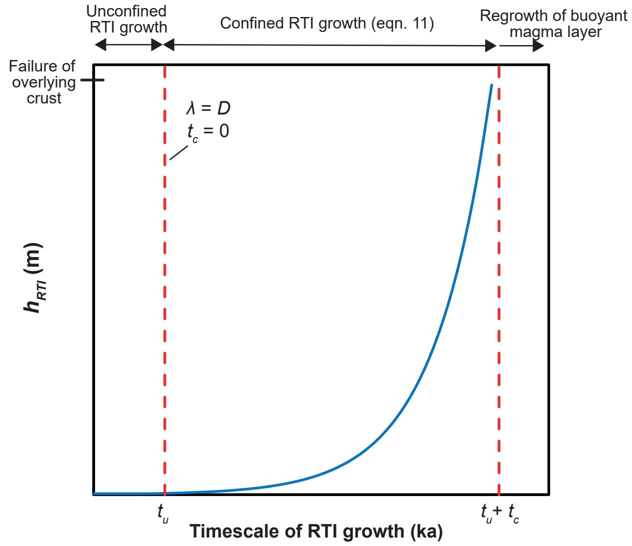

Figure S4. Timescales of Rayleigh-Taylor Instability (RTI) growth. Once a buoyant magma layer forms, the timescale t_u denotes the time of unconfined growth, during which the wavelength of the RTI increases but its amplitude h_{RTI}, remains small. When the RTI wavelength λ reaches the diameter of the reservoir ($\lambda = D$) the RTI enters the confined growth stage with timescale t_c. During this stage, the amplitude of the RTI increases exponentially (equation 11) until failure of the overlying crust (equation 9). We typically find $t_u << t_c$, so t_u is neglected in the numerical model.

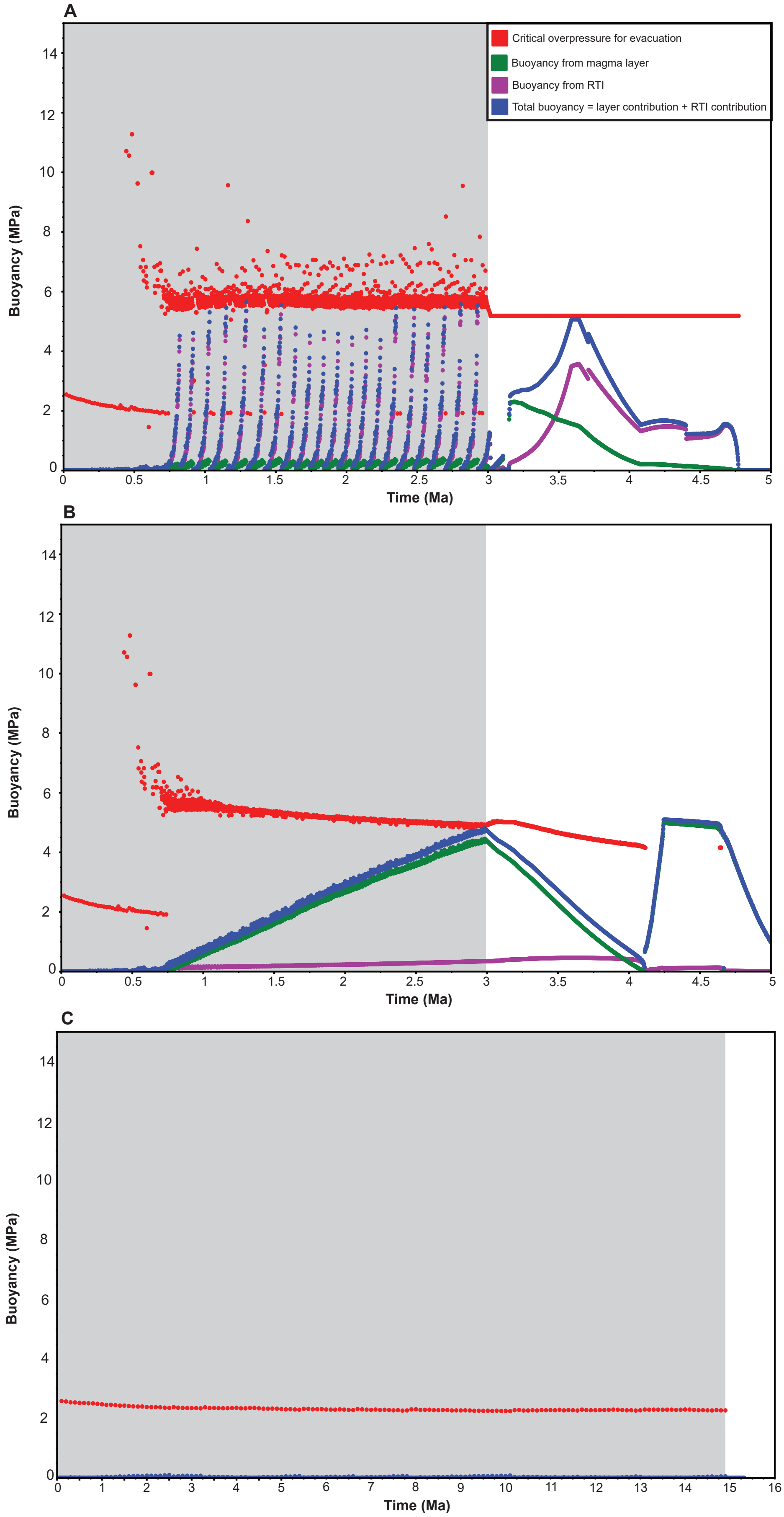

Figure S5. Evolution of buoyancy in the source reservoirs and the critical overpressure needed for evacuation. (A) Example with high dependency on RTI contribution for evacuation. The case shows has a crust shear viscosity of 10^{19} Pa.s**. (B)** Example with low dependency on RTI contribution for evacuation. The case is shown for a crust shear viscosity of 10^{21} Pa.s. **(C)** Example when no evacuations occur. The case shown is for a parameter magma intrusion flux of 5.03 km^3kyr^{-1}.The grey shading denotes the period for which sills are intruded into the reservoir.

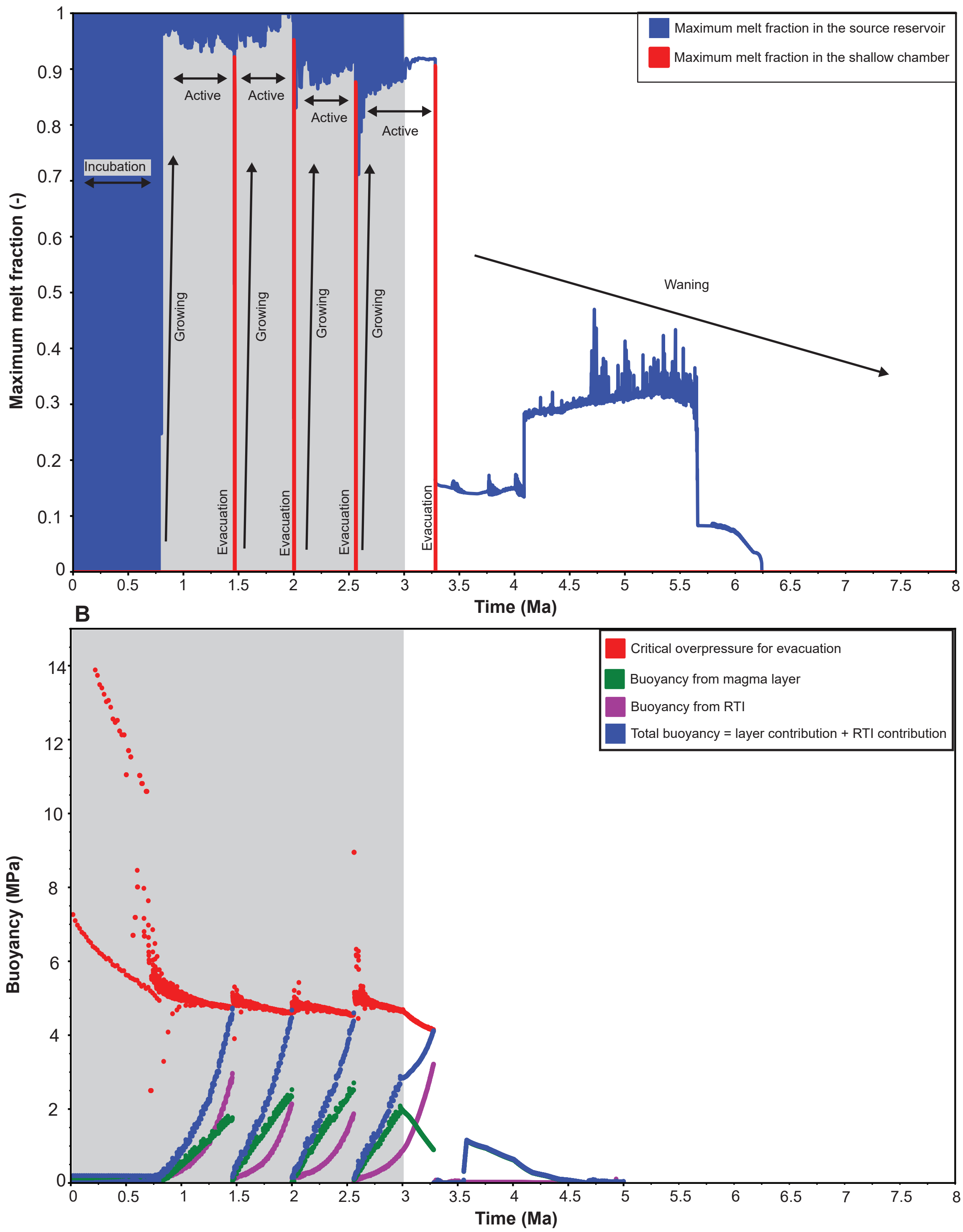

Figure S6. Evolution of (A) maximum melt fraction and (B) buoyancy in a source reservoir fed by intermediate intrusions. In this example, all inputs are the same as the example case, but intermediate sills are intruded into the crust at 20km, rather than mafic sills. The more evolved (silicic) magma accumulates more rapidly than in the example case, so there are more large evacuations from the reservoir.

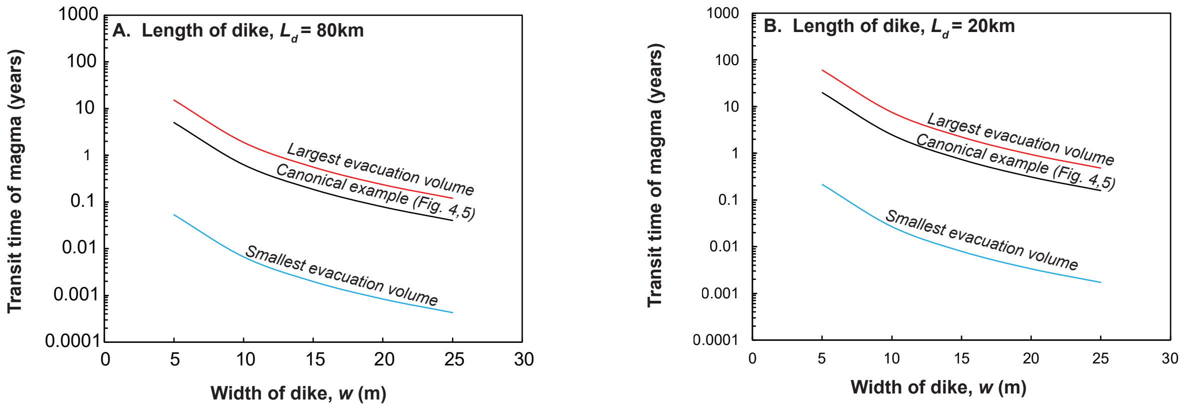

Figure S7. Transit time of evacuated silicic magma through a dike from a reservoir at 20km depth to a shallow chamber at 5km depth. Calculated transit time (volume of evacuated magma / ascent rate) for the whole volume of evacuated silicic magma as function of dike width, for two different values of dike length **(A)** 80km, equivalent to the canonical diameter of the shallow chamber, and **(B)** 20km, one quarter of the canonical shallow chamber diameter, chosen to represent a low end-member dike length. Transit times are shown for the largest and smallest volumes of silicic magma evacuated (Fig. 6) and the average volume of silicic magma evacuated in our canonical example (Fig. 4,5).

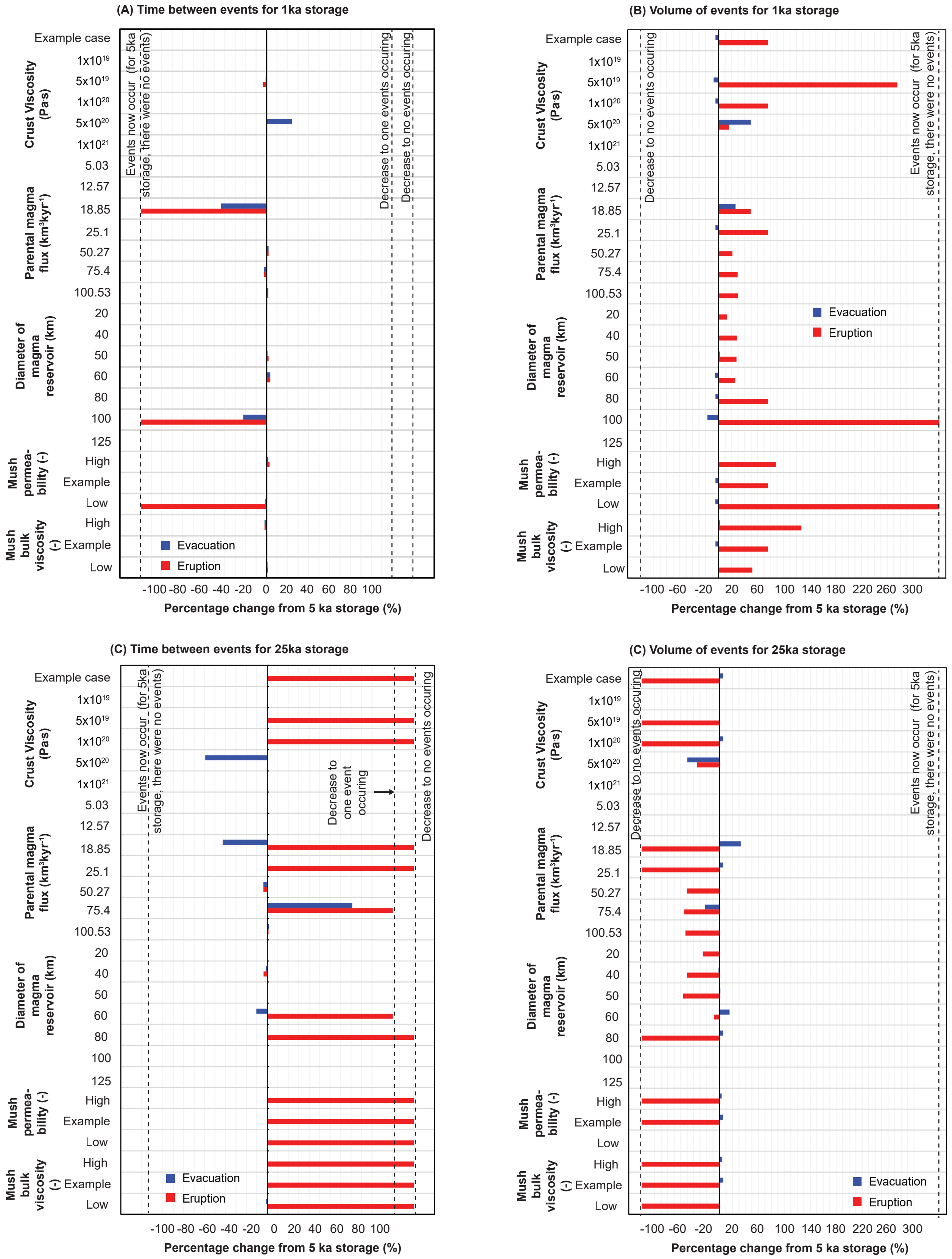

Figure S8: Percentage change in average frequency (A,C) and volume (B,D) of evacuations and eruptions for varying shallow storage timescales from 5 ka to (A,B) 1 ka and (C,D) 25 ka storage. The average composition of evacuations and eruptions varies by less than 5% for all parameters tested, so is not plotted.

Table S1: Parameters used in numerical experiments

Description and sources	Canonical case	Sensitivity analysis	Units
**Characteristic mush permeability (*33*, *53*, *73*)**	6.05×10^{-8}	1×10^{-9} - 1×10^{-7}	m^2
**Characteristic mush bulk viscosity (*33*, *53*, *72*, *73*, *83*)**	1×10^{15}	1×10^{14} - 1×10^{16}	Pa·s
**Crust shear viscosity (*45*)**	1×10^{20}	1×10^{19} - 1×10^{21}	Pa·s
**Diameter of magma reservoir**	80	25 - 125	km
**Parent magma flux (*26*, *27*, *33*)**	25.1	5.03 - 101	$km^3 \cdot kyr^{-1}$
**Freezing parameter (*4*)**	0.14	0.12 - 0.16	-
**Shear viscosity of silicic melt (Supp. Fig. 1D)**	1×10^{5}	1×10^{5}	Pa·s
**Shear viscosity of mafic melt (Supp. Fig. 1D)**	1	1	Pa·s
**Specific heat capacity (*26*, *33*, *72*, *73*)**	1100	1100	$J \cdot kg^{-1} \cdot °C^{-1}$
**Thermal conductivity (*26*, *33*, *72*, *73*)**	3	3	$W \cdot °C^{-1} \cdot m^{-1}$
**Latent heat (*26*, *33*, *72*, *73*)**	550000	550000	$J \cdot kg^{-1}$
**Elastic modulus of solid crust (*4*, *94*)**	1×10^{10}	1×10^{10}	Pa
**Parent magma sill thickness (*26*, *33*, *72*, *73*)**	0.1	0.1	km
**Density of silicic rock (Supp. Fig. 1C)**	2600	2600	$kg \cdot m^{-3}$
**Density of mafic rock (Supp. Fig. 1C)**	3000	3000	$kg \cdot m^{-3}$
**Density of silicic melt (Supp. Fig. 1C)**	2350	2350	$kg \cdot m^{-3}$
**Density of mafic melt (Supp. Fig. 1C)**	2880	2880	$kg \cdot m^{-3}$
**Critical melt fraction (*29*, *77–79*, *91*)**	0.6	0.5-0.7	-

Table S2: Data on natural eruptions plotted in Figure 10.

Volcano(source)	Location	Number of eruptions	Average bulk DRE volume erupted (km^3)	Max. bulk DRE volume erupted (km^3)	Min. bulk DRE volume erupted (km^3)	Average time between eruptions (ka)	Max. time between eruptions (ka)	Min. time between eruptions (ka)	Notes
**Maroa (*46*, *96*)**	New Zealand	9	201.64	1500.00	0.11	47.46	195.70	10.00	The volumes of the Whakamaru and Rangitawa units are combined due to possible timing overlaps (*96*)
**Apoyo(*46*)**	Nicaragua	2	16.78	31.00	2.55	0.76	0.76	0.76	
**Uzon(*46*)**	Russia	2	41.04	62.50	19.57	234.40	234.40	234.40	
**Nemo Peak(*46*)**	Kuril Islands	3	22.20	50.00	4.10	7.09	11.00	3.17	
**Ilopango(*46*)**	El Salvador	2	23.56	32.60	14.52	34.5	34.50	34.50	
**Calabozos(*46*)**	Chile	4	69.16	93.75	0.63	220.00	500.00	10.00	
**Mangakino(*19*, *46*)**	New Zealand	8	306.25	1200.00	50.00	83.57	190.00	10.00	
**Aso(*97*)**	Japan	4	212.50	600.00	50.00	58.67	125.00	18.00	
**Cerra Galán(*47*)**	Argentina	9	166.38	630.00	8.00	434.29	1210.00	40.00	
**Yellowstone(*98*, *99*)**	USA	5	768.00	2500.00	10.00	489.25	800.00	33.00	The Huckleberry Ridge Tuff unit is considered here as one unit (*98*) rather than two separate units.(*99*)
**Long Valley(*46*)**	USA	2	350.00	625.00	75.00	59.80	59.80	59.80	
**Valles(*100*)**	USA	2	400.00	400.00	400.00	350.00	350.00	350.00	
**Toba(*46*)**	Indonesia	4	1923.75	5300.00	35.00	375.00	426.00	287.00	
**Timber Mountain(*1*)**	USA	2	955.00	1100.00	810.00	200.00	200.00	200.00	
**Paintbrush(*1*)**	USA	2	1100.00	1200.00	1000.00	500.00	500.00	500.00	

Supplementary Videos:
Movie S1: Video of canonical example case (Figure 4, 5).
Movie S2: Video of case for the intrusion of intermediate magma as the parental magma (Fig. S6)

REFERENCES AND NOTES

1. B. G. Mason, D. M. Pyle, C. Oppenheimer, The size and frequency of the largest explosive eruptions on Earth. *Bull. Volcanol.* **66**, 735–748 (2004).

2. L. Caricchi, C. Annen, J. Blundy, G. Simpson, V. Pinel, Frequency and magnitude of volcanic eruptions controlled by magma injection and buoyancy. *Nat. Geosci.* **7**, 126–130 (2014).

3. S. Self, S. Blake, Consequences of explosive supereruptions. *Elements* **4**, 41–46 (2008).

4. A. M. Jellinek, D. J. DePaolo, A model for the origin of large silicic magma chambers: Precursors of caldera-forming eruptions. *Bull. Volcanol.* **65**, 363–381 (2003).

5. C. Huber, M. Townsend, W. Degruyter, O. Bachmann, Optimal depth of subvolcanic magma chamber growth controlled by volatiles and crust rheology. *Nat. Geosci.* **12**, 762–768 (2019).

6. C. J. N. Wilson, G. F. Cooper, K. J. Chamberlain, S. J. Barker, M. L. Myers, F. Illsley-Kemp, J. Farrell, No single model for supersized eruptions and their magma bodies. *Nat. Rev. Earth Environ.* **2**, 610–627 (2021).

7. A. S. R. Allan, D. J. Morgan, C. J. N. Wilson, M.-A. Millet, From mush to eruption in centuries: Assembly of the super-sized Oruanui magma body. *Contrib. Mineral. Petrol.* **166**, 143–164 (2013).

8. M. Barboni, B. Schoene, Short eruption window revealed by absolute crystal growth rates in a granitic magma. *Nat. Geosci.* **7**, 524–528 (2014).

9. M. P. Eddy, S. A. Bowring, R. B. Miller, J. H. Tepper, Rapid assembly and crystallization of a fossil large-volume silicic magma chamber. *Geology* **44**, 331–334 (2016).

10. R. L. Rudnick, Making continental crust. *Nature* **378**, 571–578 (1995).

11. T. W. Sisson, K. Ratajeski, W. B. Hankins, A. F. Glazner, Voluminous granitic magmas from common basaltic sources. *Contrib. Mineral. Petrol.* **148**, 635–661 (2005).

12. K. V. Cashman, R. S. J. Sparks, J. D. Blundy, Vertically extensive and unstable magmatic systems: A

unified view of igneous processes. *Science* **355**, eaag3055 (2017).

13. H.-H. Huang, F.-C. Lin, B. Schmandt, J. Farrell, R. B. Smith, V. C. Tsai, The Yellowstone magmatic system from the mantle plume to the upper crust. *Science* **348**, 773–776 (2015).

14. G. J. Hill, T. G. Caldwell, W. Heise, D. G. Chertkoff, H. M. Bibby, M. K. Burgess, J. P. Cull, R. A. F. Cas, Distribution of melt beneath Mount St Helens and Mount Adams inferred from magnetotelluric data. *Nat. Geosci.* **2**, 785–789 (2009).

15. K. M. Ward, G. Zandt, S. L. Beck, D. H. Christensen, H. McFarlin, Seismic imaging of the magmatic underpinnings beneath the Altiplano-Puna volcanic complex from the joint inversion of surface wave dispersion and receiver functions. *Earth Planet. Sci. Lett.* **404**, 43–53 (2014).

16. W. Degruyter, C. Huber, A model for eruption frequency of upper crustal silicic magma chambers. *Earth Planet. Sci. Lett.* **403**, 117–130 (2014).

17. T. H. Druitt, F. Costa, E. Deloule, M. Dungan, B. Scaillet, Decadal to monthly timescales of magma transfer and reservoir growth at a caldera volcano. *Nature* **482**, 77–80 (2012).

18. A. Burgisser, G. W. Bergantz, A rapid mechanism to remobilize and homogenize highly crystalline magma bodies. *Nature* **471**, 212–215 (2011).

19. K. M. Cooper, A. J. R. Kent, Rapid remobilization of magmatic crystals kept in cold storage. *Nature* **506**, 480–483 (2014).

20. D. Szymanowski, J.-F. Wotzlaw, B. S. Ellis, O. Bachmann, M. Guillong, A. von Quadt, Protracted near-solidus storage and pre-eruptive rejuvenation of large magma reservoirs. *Nat. Geosci.* **10**, 777–782 (2017).

21. W. J. Malfait, R. Seifert, S. Petitgirard, J.-P. Perrillat, M. Mezouar, T. Ota, E. Nakamura, P. Lerch, C. Sanchez-Valle, Supervolcano eruptions driven by melt buoyancy in large silicic magma chambers. *Nat. Geosci.* **7**, 122–125 (2014).

22. P. M. Gregg, S. L. De Silva, E. B. Grosfils, J. P. Parmigiani, Catastrophic caldera-forming eruptions:

Thermomechanics and implications for eruption triggering and maximum caldera dimensions on Earth. *J. Volcanol. Geotherm. Res.* **241–242**, 1–12 (2012).

23. A. S. R. Allan, C. J. N. Wilson, M.-A. Millet, R. J. Wysoczanski, The invisible hand: Tectonic triggering and modulation of a rhyolitic supereruption. *Geology* **40**, 563–566 (2012).

24. P. M. Gregg, E. B. Grosfils, S. L. De Silva, Catastrophic caldera-forming eruptions II: The subordinate role of magma buoyancy as an eruption trigger. *J. Volcanol. Geotherm. Res.* **305**, 100–113 (2015).

25. H. E. Cabaniss, P. M. Gregg, E. B. Grosfils, The role of tectonic stress in triggering large silicic caldera eruptions. *Geophys. Res. Lett.* **45**, 3889–3895 (2018).

26. C. Annen, J. D. Blundy, R. S. J. Sparks, The genesis of intermediate and silicic magmas in deep crustal hot zones. *J. Petrol.* **47**, 505–539 (2006).

27. C. Annen, From plutons to magma chambers: Thermal constraints on the accumulation of eruptible silicic magma in the upper crust. *Earth Planet. Sci. Lett.* **284**, 409–416 (2009).

28. O. Karakas, W. Degruyter, O. Bachmann, J. Dufek, Lifetime and size of shallow magma bodies controlled by crustal-scale magmatism. *Nat. Geosci.* **10**, 446–450 (2017).

29. B. D. Marsh, On the crystallinity, probability of occurrence, and rheology of lava and magma. *Contrib. Mineral. Petrol.* **78**, 85–98 (1981).

30. A. R. Philpotts, M. Carroll, Physical properties of partly melted tholeiitic basalt. *Geology* **24**, 1029–1032 (1996).

31. A. Costa, L. Caricchi, N. Bagdassarov, A model for the rheology of particle-bearing suspensions and partially molten rocks. *Geochem. Geophys. Geosyst.* **10**, 10.1029/2008GC002138 (2009).

32. N. Petford, R. C. Kerr, J. R. Lister, Dike transport of granitoid magmas. *Geology* **21**, 845–848 (1993).

33. M. D. Jackson, J. Blundy, R. S. J. Sparks, Chemical differentiation, cold storage and remobilization of magma in the Earth's crust. *Nature* **564**, 405–409 (2018).

34. M. D. Jackson, M. J. Cheadle, A continuum model for the transport of heat, mass and momentum in a deformable, multicomponent mush, undergoing solid-liquid phase change. *Int. J. Heat Mass Transf.* **41**, 1035–1048 (1998).

35. H. Hu, M. D. Jackson, J. Blundy, Melting, compaction and reactive flow: Controls on melt fraction and composition change in crustal mush reservoirs. *J. Petrol.* **63**, egac097 (2022).

36. R. F. Weinberg, Y. Podladchikov, Diapiric ascent of magmas through power law crust and mantle. *J. Geophys. Res.* **99**, 9543–9559 (1994).

37. A. F. Glazner, J. M. Bartley, D. S. Coleman, W. Gray, R. Z. Taylor, Are plutons assembled over millions of years by amalgamation from small magma chambers? *GSA Today* **14**, 4 (2004).

38. C. F. Miller, D. J. Furbish, B. A. Walker, L. L. Claiborne, G. C. Koteas, H. A. Bleick, J. S. Miller, Growth of plutons by incremental emplacement of sheets in crystal-rich host: Evidence from Miocene intrusions of the Colorado River region, Nevada, USA. *Tectonophysics* **500**, 65–77 (2011).

39. G. Seropian, A. C. Rust, R. S. J. Sparks, The gravitational stability of lenses in magma mushes: Confined Rayleigh-Taylor instabilities. *J. Geophys. Res. Solid Earth* **123**, 3593–3607 (2018).

40. T. Menand, Physical controls and depth of emplacement of igneous bodies: A review. *Tectonophysics* **500**, 11–19 (2011).

41. R. S. J. Sparks, C. Annen, J. D. Blundy, K. V. Cashman, A. C. Rust, M. D. Jackson, Formation and dynamics of magma reservoirs. *Phil. Trans. R. Soc. A.* **377**, 20180019 (2019).

42. H. Hu, P. Salinas, M. Jackson, "A 3D finite element magma reservoir simulator" (Vienna, 2023; https://meetingorganizer.copernicus.org/EGU23/EGU23-5441.html).

43. H. Hu, P. Salinas, M. D. Jackson, "V32E-0108 IC-FEMRES: A new 3D two-phase finite element magma reservoir simulator." (Chicago, USA, 2022).

44. M. J. Comeau, M. J. Unsworth, F. Ticona, M. Sunagua, Magnetotelluric images of magma distribution beneath Volcán Uturuncu, Bolivia: Implications for magma dynamics. *Geology* **43**, 243–246 (2015).

45. R. Bürgmann, G. Dresen, Rheology of the lower crust and upper mantle: Evidence from rock mechanics, geodesy, and field observations. *Annu. Rev. Earth Planet. Sci.* **36**, 531–567 (2008).

46. H. S. Crosweller, B. Arora, S. K. Brown, E. Cottrell, N. I. Deligne, N. O. Guerrero, L. Hobbs, K. Kiyosugi, S. C. Loughlin, J. Lowndes, M. Nayembil, L. Siebert, R. S. J. Sparks, S. Takarada, E. Venzke, Global database on large magnitude explosive volcanic eruptions (LaMEVE). *J Appl. Volcanol.* **1**, 4 (2012).

47. C. B. Folkes, H. M. Wright, R. A. F. Cas, S. L. de Silva, C. Lesti, J. G. Viramonte, A re-appraisal of the stratigraphy and volcanology of the Cerro Galán volcanic system, NW Argentina. *Bull. Volcanol.* **73**, 1427–1454 (2011).

48. E. Cañón-Tapia, Volcanic eruption triggers: A hierarchical classification. *Earth-Sci. Rev.* **129**, 100–119 (2014).

49. L. Caricchi, T. E. Sheldrake, J. Blundy, Modulation of magmatic processes by CO_2 flushing. *Earth Planet. Sci. Lett.* **491**, 160–171 (2018).

50. M. Townsend, C. Huber, A critical magma chamber size for volcanic eruptions. *Geology* **48**, 431–435 (2020).

51. T. H. Druitt, M. Mercier, L. Florentin, E. Deloule, N. Cluzel, T. Flaherty, E. Médard, A. Cadoux, Magma Storage and Extraction Associated with Plinian and Interplinian Activity at Santorini Caldera (Greece). *J. Petrol.* **57**, 461–494 (2016).

52. M. Edmonds, A. W. Woods, Exsolved volatiles in magma reservoirs. *J. Volcanol. Geotherm. Res.* **368**, 13–30 (2018).

53. J. M. S. Solano, M. D. Jackson, R. S. J. Sparks, J. Blundy, Evolution of major and trace element composition during melt migration through crystalline mush: Implications for chemical differentiation in the crust. *Am. J. Sci.* **314**, 895–939 (2014).

54. F. Gutiérrez, M. A. Parada, Numerical modeling of time-dependent fluid dynamics and differentiation of a shallow basaltic magma chamber. *J. Petrol.* **51**, 731–762 (2010).

55. H. Schmeling, G. Marquart, R. Weinberg, H. Wallner, Modelling melting and melt segregation by two-phase flow: New insights into the dynamics of magmatic systems in the continental crust. *Geophys. J. Int.* **217**, 422–450 (2019).

56. Y.-Q. Wong, T. Keller, A unified numerical model for two-phase porous, mush and suspension flow dynamics in magmatic systems. *Geophys. J. Int.* **233**, 769–795 (2023).

57. D. Vielzeuf, J. M. Montel, Partial melting of metagreywackes. Part I. Fluid-absent experiments and phase relationships. *Contrib. Mineral. Petrol.* **117**, 375–393 (1994).

58. D. L. Blatter, T. W. Sisson, W. B. Hankins, Crystallization of oxidized, moderately hydrous arc basalt at mid- to lower-crustal pressures: Implications for andesite genesis. *Contrib. Mineral. Petrol.* **166**, 861–886 (2013).

59. Y. Bottinga, D. F. Weill, The viscoisty of magmatic silicate liquids: A model for calculation. *Am. J. Sci.* **272**, 438–475 (1972).

60. X. Guo, R. A. Lange, Y. Ai, Density and sound speed measurements on model basalt (An–Di–Hd) liquids at one bar: New constraints on the partial molar volume and compressibility of the FeO component. *Earth Planet. Sci. Lett.* **388**, 283–292 (2014).

61. M. Hartley, J. Maclennan, Magmatic densities control erupted volumes in icelandic volcanic systems. *Front. Earth Sci.* **6**, 29 (2018).

62. T. Murase, A. R. McBirney, Properties of some common igneous rocks and their melts at high temperatures. *Geol. Soc. Am. Bull.* **84**, 3563–3592 (1973).

63. F. A. Ochs III, R. A. Lange, The density of hydrous magmatic liquids. *Science* **283**, 1314–1317 (1999).

64. S. Sinigoi, J. E. Quick, A. Mayer, G. Demarchi, Density-controlled assimilation of underplated crust, Ivrea-Verbano zone, Italy. *Earth Planet. Sci. Lett.* **129**, 183–191 (1995).

65. E. Stolper, D. Walker, Melt density and the average composition of basalt. *Contrib. Mineral. Petrol.* **74**, 7–12 (1980).

66. D. B. Dingwell, C. Romano, K.-U. Hess, The effect of water on the viscosity of a haplogranitic melt under *P-T-X* conditions relevant to silicic volcanism. *Contrib. Mineral. Petrol.* **124**, 19–28 (1996).

67. D. Giordano, D. Dingwell, Viscosity of hydrous Etna basalt: Implications for Plinian-style basaltic eruptions. *Bull. Volcanol.* **65**, 8–14 (2003).

68. D. Giordano, J. K. Russell, D. B. Dingwell, Viscosity of magmatic liquids: A model. *Earth Planet. Sci. Lett.* **271**, 123–134 (2008).

69. E. Hartung, G. Weber, L. Caricchi, The role of H_2O on the extraction of melt from crystallising magmas. *Earth Planet. Sci. Lett.* **508**, 85–96 (2019).

70. H. R. Shaw, Viscosities of magmatic silicate liquids: An empirical method of prediction. *Am. J. Sci.* **272**, 870–893 (1972).

71. A. Whittington, P. Richet, H. Behrens, F. Holtz, B. Scaillet, Experimental temperature-X(H_2O)-viscosity relationship for leucogranites and comparison with synthetic silicic liquids. *Earth Environ. Sci. Trans. R. Soc. Edinb.* **95**, 59–71 (2004).

72. J. A. D. Connolly, Y. Y. Podladchikov, Compaction-driven fluid flow in viscoelastic rock. *Geodin. Acta* **11**, 55–84 (1998).

73. M. D. Jackson, M. J. Cheadle, M. P. Atherton, Quantitative modeling of granitic melt generation and segregation in the continental crust. *J. Geophys. Res.* **108**, 10.1029/2001JB001050 (2003).

74. M. B. Holness, M. A. Hallworth, A. Woods, R. E. Sides, Infiltration metasomatism of cumulates by intrusive magma replenishment: The Wavy Horizon, Isle of Rum, Scotland. *J. Petrol.* **48**, 563–587 (2007).

75. R. Maghdour-Mashhour, B. Hayes, R. Bolhar, H. Ueckermann, Sill intrusion into pyroxenitic mush and the development of the Lower–Upper Critical Zone boundary of the Bushveld Complex: Implications for the origin of stratiform anorthosites and chromitites in layered intrusions. *J. Petrol.* **62**, egaa090 (2021).

76. J. E. Mungall, S. L. Kamo, S. McQuade, U–Pb geochronology documents out-of-sequence emplacement of ultramafic layers in the Bushveld Igneous Complex of South Africa. *Nat. Commun.* **7**, 13385 (2016).

77. L. Caricchi, L. Burlini, P. Ulmer, T. Gerya, M. Vassalli, P. Papale, Non-Newtonian rheology of crystal-bearing magmas and implications for magma ascent dynamics. *Earth Planet. Sci. Lett.* **18**, 402–419 (2007).

78. R. Champallier, M. Bystricky, L. Arbaret, Experimental investigation of magma rheology at 300MPa: From pure hydrous melt to 76vol.% of crystals. *Earth Planet. Sci. Lett.* **267**, 571–583 (2008).

79. A.-M. Lejeune, P. Richet, Rheology of crystal-bearing silicate melts: An experimental study at high viscosities. *J. Geophys. Res. Solid Earth* **100**, 4215–4229 (1995).

80. C. L. Rosenberg, M. R. Handy, Experimental deformation of partially melted granite revisited: Implications for the continental crust. *J. Metamorph. Geol.* **23**, 19–28 (2005).

81. T. Keller, J. Suckale, A continuum model of multi-phase reactive transport in igneous systems. *Geophys. J. Int.* **219**, 185–222 (2019).

82. J. A. D. Connolly, M. W. Schmidt, Viscosity of crystal-mushes and implications for compaction-driven fluid flow. *J. Geophys. Res. Solid Earth* **127**, e2022JB024743 (2022).

83. H. Schmeling, J. P. Kruse, G. Richard, Effective shear and bulk viscosity of partially molten rock based on elastic moduli theory of a fluid filled poroelastic medium. *Geophys. J. Int.* **190**, 1571–1578 (2012).

84. G. W. Bergantz, J. M. Schleicher, A. Burgisser, Open-system dynamics and mixing in magma mushes. *Nat. Geosci.* **8**, 793–796 (2015).

85. S. Couch, R. S. J. Sparks, M. R. Carroll, Mineral disequilibrium in lavas explained by convective self-mixing in open magma chambers. *Nature* **411**, 1037–1039 (2001).

86. C. Huber, O. Bachmann, M. Manga, Homogenization processes in silicic magma chambers by stirring and mushification (latent heat buffering). *Earth Planet. Sci. Lett.* **283**, 38–47 (2009).

87. D. Floess, L. Caricchi, G. Simpson, S. R. Wallis, Melt segregation and the architecture of magmatic reservoirs: Insights from the Muroto sill (Japan). *Contrib. Mineral. Petrol.* **174**, 27 (2019).

88. F. G. F. Gibb, C. M. B. Henderson, Chemistry of the Shiant Isles Main Sill, NW Scotland, and Wider Implications for the Petrogenesis of Mafic Sills. *J. Petrol.* **47**, 191–230 (2006).

89. R. M. Latypov, The origin of basic-ultrabasic sills with S-, D-, and I-shaped compositional profiles by in situ crystallization of a single input of phenocryst-poor parental magma. *J. Petrol.* **44**, 1619–1656 (2003).

90. A. Gudmundsson, Magma chambers: Formation, local stresses, excess pressures, and compartments. *J. Volcanol. Geotherm. Res.* **237–238**, 19–41 (2012).

91. F. Sigmundsson, V. Pinel, R. Grapenthin, A. Hooper, S. A. Halldórsson, P. Einarsson, B. G. Ófeigsson, E. R. Heimisson, K. Jónsdóttir, M. T. Gudmundsson, K. Vogfjörd, M. Parks, S. Li, V. Drouin, H. Geirsson, S. Dumont, H. M. Fridriksdottir, G. B. Gudmundsson, T. J. Wright, T. Yamasaki, Unexpected large eruptions from buoyant magma bodies within viscoelastic crust. *Nat. Commun.* **11**, 2403 (2020).

92. J. de Bremond d'Ars, C. Jaupart, R. S. J. Sparks, Distribution of volcanoes in active margins. *J. Geophys. Res. Solid Earth* **100**, 20421–20432 (1995).

93. J. A. Whitehead Jr., D. S. Luther, Dynamics of laboratory diapir and plume models. *J. Geophys. Res. Solid Earth* **80**, 705–717 (1975).

94. A. M. Rubin, Getting granite dikes out of the source region. *J. Geophys. Res. Solid Earth* **100**, 5911–5929 (1995).

95. N. Petford, J. R. Lister, R. C. Kerr, The ascent of felsic magmas in dykes. *Lithos* **32**, 161–168 (1994).

96. D. T. Downs, C. J. N. Wilson, J. W. Cole, J. V. Rowland, A. T. Calvert, G. S. Leonard, J. M. Keall, Age and eruptive center of the Paeroa Subgroup ignimbrites (Whakamaru Group) within the Taupo Volcanic Zone of New Zealand. *Geol. Soc. Am. Bull.* **126**, 1131–1144 (2014).

97. K. Kaneko, H. Kamata, T. Koyaguchi, M. Yoshikawa, K. Furukawa, Repeated large-scale eruptions

from a single compositionally stratified magma chamber: An example from Aso volcano, Southwest Japan. *J. Volcanol. Geotherm. Res.* **167**, 160–180 (2007).

98. J.-F. Wotzlaw, I. N. Bindeman, R. A. Stern, F.-X. D'Abzac, U. Schaltegger, Rapid heterogeneous assembly of multiple magma reservoirs prior to Yellowstone supereruptions. *Sci. Rep.* **5**, 14026 (2015).

99. B. S. Ellis, D. F. Mark, C. J. Pritchard, J. A. Wolff, Temporal dissection of the Huckleberry Ridge Tuff using the $^{40}Ar/^{39}Ar$ dating technique. *Quat. Geochronol.* **9**, 34–41 (2012).

100. J. Wu, S. J. Cronin, M. C. Rowe, J. A. Wolff, S. J. Barker, B. Fu, S. Boroughs, Crustal evolution leading to successive rhyolitic supereruptions in the Jemez Mountains volcanic field, New Mexico, USA. *Lithos* **396–397**, 106201 (2021).
